# Role of Cinchona Alkaloids in the Enantio- and Diastereoselective Synthesis of Axially Chiral Compounds

**DOI:** 10.1021/acs.accounts.2c00515

**Published:** 2022-12-07

**Authors:** Chiara Portolani, Giovanni Centonze, Paolo Righi, Giorgio Bencivenni

**Affiliations:** † Department of Industrial Chemistry “Toso Montanari,” Alma Mater Studiorum−University of Bologna, viale del Risorgimento 4, 40136 Bologna, Italy; ‡ Centre for the Chemical Catalysis−C^3^, Alma Mater Studiorum−University of Bologna, viale del Risorgimento 4, 40136 Bologna, Italy

## Abstract

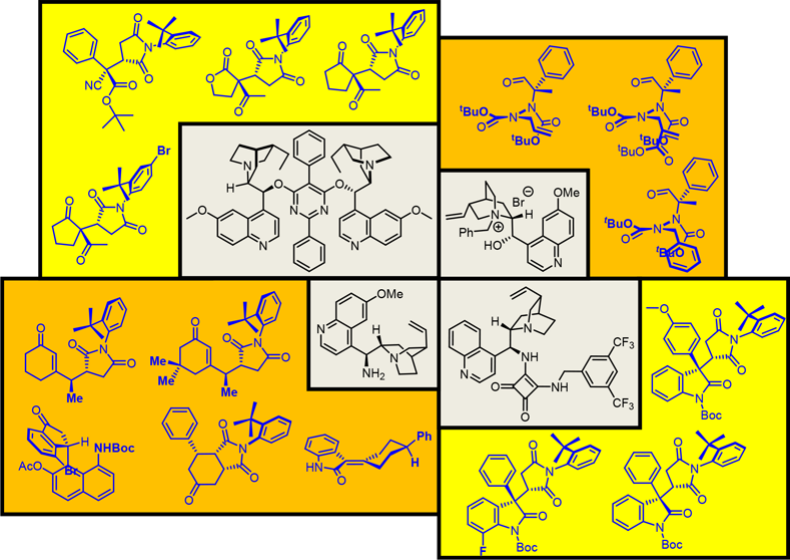

Asymmetric synthesis using organic catalysts has evolved since it was first realized and defined. Nowadays, it can be considered a valid alternative to transition metal catalysis for synthesizing chiral molecules. According to the literature, the number of asymmetric organocatalytic processes associated with atropisomer synthesis has rapidly increased over the past 10 years because organocatalysis addresses the challenges posed by the most widespread strategies used for preparing axially chiral molecules with satisfactory results.

These strategies, useful to prepare a wide range of C–C, C–heteroatom, and N–N atropisomers, vary from kinetic resolution to direct arylation, desymmetrization, and central-to-axial chirality conversion. In this field, our contribution focuses on determining novel methods for synthesizing atropisomers, during which, in most cases, the construction of one or more stereogenic centers other than the stereogenic axis occurred. To efficiently address this challenge, we exploited the ability of catalysts based on a cinchona alkaloid scaffold to realize enantioselective organic transformations. Desymmetrization of *N*-(2-*tert*-butylphenyl) maleimides was one of the first strategies that we pursued for preparing C–N atropisomers. The main principle is based on the presence of a rotationally hindered C–N single bond owing to the presence of a large *tert*-butyl group. Following the peculiar reactivity of this type of substrate as a powerful electrophile and dienophile, we realized several transformations.

First, we investigated the vinylogous Michael addition of 3-substituted cyclohexenones, where a stereogenic axis and two contiguous stereocenters were concomitantly and remotely formed and stereocontrolled using a primary amine catalyst. Subsequently, we realized desymmetrization via an organocatalytic Diels–Alder reaction of activated unsaturated ketones that enabled highly atropselective transformation with efficient diastereoselectivity, thereby simultaneously controlling four stereogenic elements. Employing chiral organic bases allowed us to realize efficient desymmetrizations using carbon nucleophiles, such as 1,3-dicarbonyl compounds, cyanoacetates, and oxindoles. These reactions, performed with different types of catalysts, highlighted the versatility of organocatalysis as a powerful strategy for atropselective desymmetrization of pro-axially chiral maleimides.

Hereafter, we studied the Friedel–Crafts alkylation of naphthols with indenones, a powerful method for enantioselective synthesis of conformationally restricted diastereoisomeric indanones. We realized the first axially chiral selective Knoevenagel condensation using cinchona alkaloid primary amine as the catalyst. This reaction provided a powerful method to access enantioenriched olefins containing the oxindole core. Subsequently, we initiated an intense program for the computational investigation of the reaction mechanism of our atropselective processes. An understanding of the catalytic activity for vinylogous atropselective desymmetrization as well as of the role played by the acidic cocatalyst used for the experimental work was achieved.

Recently, we have garnered interest in the novel frontiers of atropselective synthesis. As observed in recent publications, there is considerable interest in the development of methods for preparing N–N atropisomers, an emerging topic in the field of atropselective synthesis. We focused on the synthesis of hydrazide atropisomers by developing a one-pot sequential catalysis protocol based on two sequential organocatalytic reactions that provided high stereocontrol of two contiguous stereogenic elements.

## Key References

- Di IorioN.; RighiP.; MazzantiA.; MancinelliM.; CiogliA.; BencivenniG.Remote Control of Axial Chirality: Aminocatalytic Desymmetrization of *N*-Arylmaleimides via Vinylogous Michael Addition. J. Am. Chem. Soc.2014, 136, 102502500698410.1021/ja505610k.^1^*This study demonstrates the efficiency of cinchona alkaloid primary amine to desymmetrize rotationally hindered maleimides via Michael addition of vinylogous intermediates for realizing the first organocatalytic enantioselective access to atropisomeric succinimides.*
- CrottiS.; Di IorioN.; ArtusiC.; MazzantiA.; RighiP.; BencivenniG.Direct Access to Alkylideneoxindoles via Axially Enantioselective Knoevenagel Condensation. Org. Lett.2019, 21, 30133097766210.1021/acs.orglett.9b00505.^2^*This study demonstrates the efficiency of cinchona alkaloid primary amine to synthesize axially chiral alkylideneoxindoles via E1cb elimination pathway. The process represents a useful strategy for realizing enantioselective olefination reactions.*
- PortolaniC.; CentonzeG.; LucianiS.; PellegriniA.; RighiP.; MazzantiA.; CiogliA.; SoratoA.; BencivenniG.Synthesis of Atropisomeric Hydrazides by One-Pot Sequential Enantio- and Diastereoselective Catalysis. Angew. Chem., Int. Ed.2022, 61, e20220989510.1002/anie.202209895PMC982627036036383.^3^*This study demonstrates the effective use of sequential catalysis in the enantio- and diastereoselective synthesis of a novel class of N–N atropisomers. The catalyst permutation enables the synthesis of diastereoisomers in a stereodivergent manner.*

## 1 Introduction

Asymmetric synthesis is an excellent method to selectively prepare chiral scaffolds. The high demand for methods to synthesize chiral molecules is driven by the fact that chirality is a fundamental property of bioactive natural products, drugs, and catalysts. Molecules that possess stereogenic carbon centers play a vital role in this field. However, in the past 15 years, molecules characterized by a form of chirality originating from restricted rotations along chemical single bonds, i.e., atropisomers, attracted several research groups owing to their innate properties; these molecules act as efficient ligands for asymmetric synthesis catalyzed by transition metals.^4^ Famous ligands, such as BINAM, BINOL, or BINAP, represent core structures at the bases of complicated architectures designed and perfected over the years to improve the efficiency of enantioselective catalytic processes. However, atropisomers are not limited to ligands and catalysts. Owing to the abundant availability of natural compounds that display axial chirality,^5^ several researchers have focused on preparing atropisomers as target compounds. Currently, enantioselective synthesis of atropisomers can be considered a mature field in the asymmetric synthesis scenario. The effectiveness of atroposelective transformations depends on two requirements to be met at the same time: (1) the rotational stability of the reaction product, which is, in most cases, the result of large steric hindrance around the axis of chirality, and (2) control over stereochemistry exerted by the catalyst when sterically hindered substrates are used. The first requirement is an intrinsic aspect of atropselective transformations because without steric hindrance, rotational stability cannot be achieved. However, this aspect poses a challenge for new atropisomers and can be satisfied by deploying sterically encumbered reaction partners. The second requirement represents the main core of any asymmetric synthesis; however, owing to the twisted conformation of atropisomers, appropriately designed multifunctional catalysts that can simultaneously coordinate reaction partners, giving a twisted imprint to the stereodetermining transition state reaction, are required. Chiral phosphoric acids, short peptides, and cinchona alkaloids are good promoters of atropselective transformations. Effective catalytic tactics and strategies were reported with regard to the use of organo-based or transition metal based catalysts.^6^ Further, C(sp^2^)–C(sp^2^) atropisomers, which have been the base for the first studies on atropisomerism, are considered important. Among diffuse atropisomers, biaryls are characterized by high rotational energy barriers with values >30 kcal/mol, which endow them with high stereochemical stability.^7^ Moreover, a rapid escalation of different classes of atropisomeric compounds has been observed over the past 10 years. Accordingly, C–S, C–O, and specifically C–N atropisomers have been investigated in detail.^8^ Particularly, C–N atropisomers represent an important category of stereoisomers and are often found in biologically active natural products, for which the hindered C–N single bond is the key stereogenic element that underlies their biological properties. Most strategies employed to accomplish the stereoselective preparation of atropisomers are based on dynamic kinetic resolution and desymmetrization. However, in recent years, novel reactions have been reported as direct synthesis strategies, in which the stereogenic axis is generated in a single chemical operation. Currently, coupling reactions, chirality conversion, arylation, and arene-forming reactions are considered to be the most versatile synthesis methods.^9^ Herein, we report the results of asymmetric synthesis of atropisomers using organic catalysts based on the cinchona alkaloid scaffolds, which can help exert the tridimensional arrangement of the chiral axis. We highlight how these chiral organocatalysts can control the stereogenic axis and concomitant presence of more than one stereogenic center exploiting their ability to interact with various substrates through different activation modes ranging from covalent enamine/iminium ion catalysis to counterion-directed catalysis through hydrogen bonding and π-stacking interactions. The versatility expressed and possibility to be easily functionalized make these commercially available catalysts a mild alternative to metal-based catalysts.

## 2 Desymmetrization Strategy for Synthesizing C–N Atropisomers

**Scheme 1:**
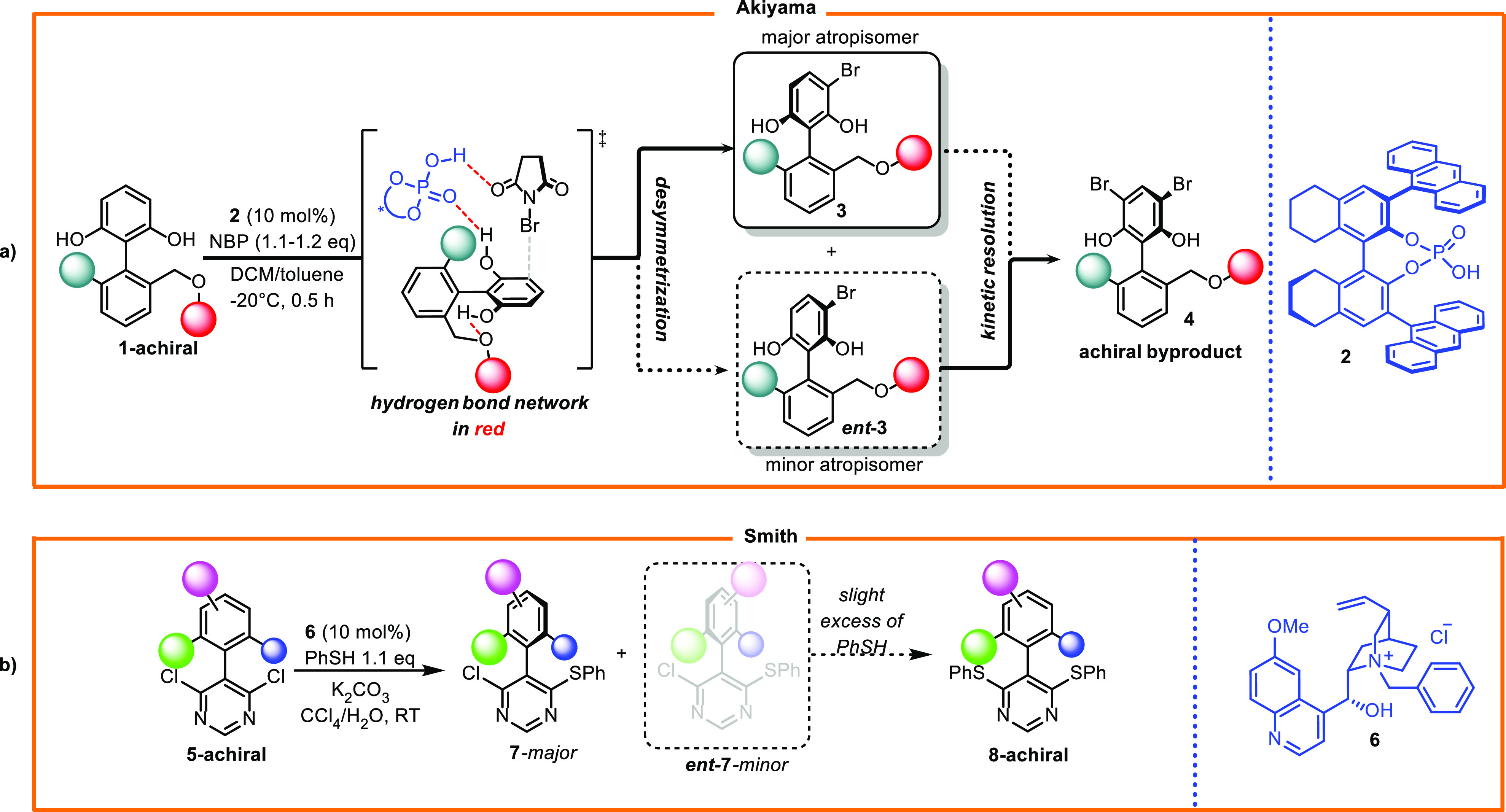
(a) Atropselective Desymmetrization of Tetrasubstituted Biaryls and (b) Atropselective Synthesis of Pyrimidine

Among organocatalytic strategies that allow one to build a stereogenic axis, desymmetrization reactions occur on prochiral or meso-compounds, which possess a rotationally constrained single bond.^10^ Therefore, the stereoselective symmetry break is accomplished if the chiral catalyst can distinguish between the two atropotopic faces of the substrate, which originate from the plane of symmetry containing the axis. The first organocatalytic reactions demonstrated desymmetrization of biaryls and were performed using different strategies based on electrophilic and nucleophilic aromatic substitution reactions. In 2013, Akiyama et al. reported the selective bromination of tetrasubstituted biaryls catalyzed by chiral phosphoric acid (Scheme 1a).^11^ The reaction was performed on 2′-(alkoxymethyl)-[1,1′-biphenyl]-2,6-diol **1**, which was carefully designed, in which two hydroxy groups established intramolecular and intermolecular hydrogen bonds, which are beneficial for the reaction in terms of reactivity and stereoselectivity. Catalyst **2** promoted bromination was realized through the concomitant activation of electrophilic *N*-bromosuccinimide and nucleophilic aromatic diols in a highly organized cyclic transition state, thus ensuring excellent enantiocontrol.

In 2014, Smith developed a technique for desymmetrization of dichloropyrimidines **5** based on an organocatalytic nucleophilic aromatic substitution mechanism (Scheme 1b).^12^ The reaction was performed using a phase transfer catalyst that promoted addition of thiophenol anions to the activated pyrimidine core. *N*-Benzylquininium chloride **6** efficiently desymmetrized substituted dichloropyrimidines in a highly selective manner. In the aforementioned cases, kinetic resolution occurring during the reaction increased the enantiomeric excess, thereby releasing a negligible amount of the achiral product and almost enantiopure axially chiral biaryl.

**Scheme 2:**
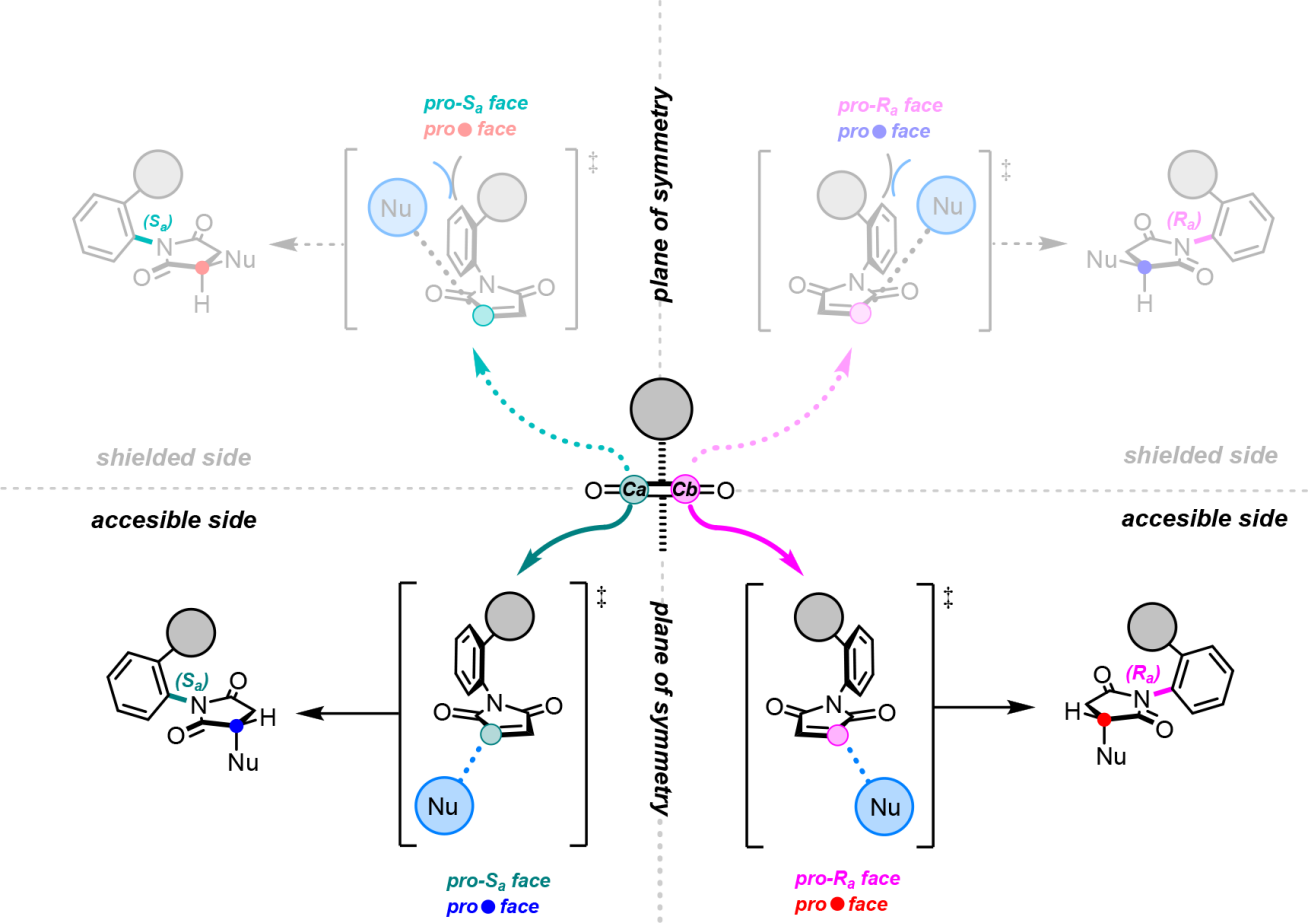
Behavior of *N*-Arylmaleimide in Desymmetrization Reactions

The first case of organocatalytic desymmetrization via vinylogous Michael addition to *N*-(2-*tert*-butylphenyl)maleimides, which led to the formation of C–N atropisomeric succinimides, was reported by us in 2014.^1^ Maleimides have been widely used in different organic reactions owing to the strong electrophilicity of their double bond.^13^ Additionally, the corresponding succinimide is an important moiety found in pharmaceutical and biological compounds.^14^ Before our organocatalytic approach, preliminary studies conducted by Curran et al. on alkyl radical addition to *N*-arylmaleimides were fundamental to comprehend the interesting features of these compounds.^15^ Particularly, a bulky substituent placed at the ortho position of the phenyl ring demonstrates two main effects: (1) It limits the free rotation around the N–C bond, thus creating a plane of symmetry that bisects the substrate; (2) It shields one side of the maleimide from a nucleophilic attack via steric hindrance (Scheme 2).

**Scheme 3:**
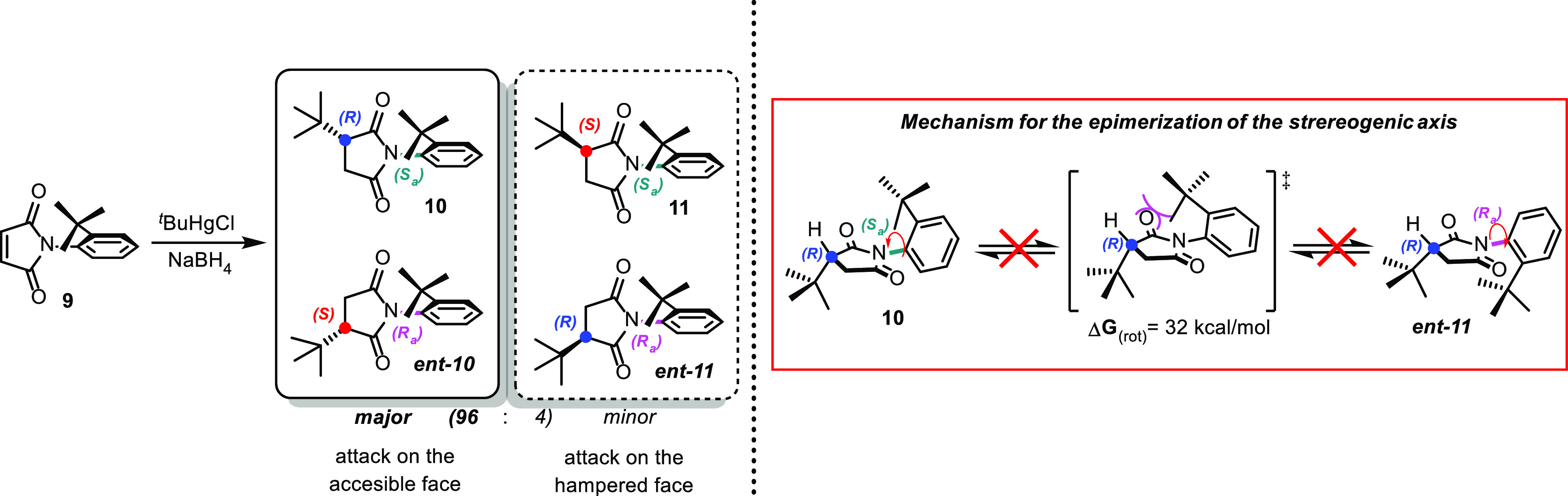
Effect of *tert*-Butyl on Hampering the Access to One Side of Maleimide

These aforementioned effects were best expressed in the Giese reaction of *N*-(2-*tert*-butylphenyl)maleimide **9** that directed the *tert*-butyl radical to the face not shielded by the ortho substituent, thus forming a racemic mixture of one prevailing diastereoisomer (Scheme 3).

**Scheme 4:**
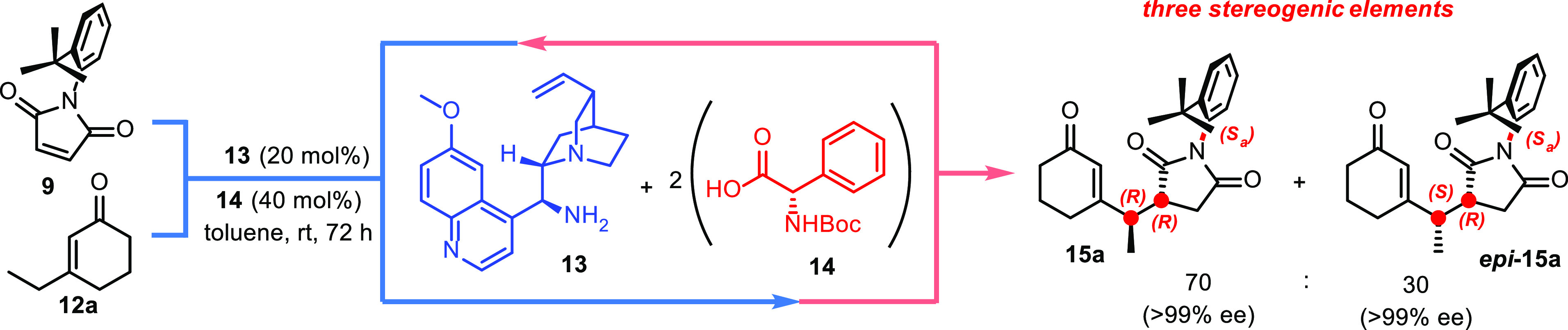
Organocatalytic Strategy for the Stereoselective Vinylogous Michael Addition

We performed a desymmetrization reaction using an organocatalyst, which can recognize the atropotopic faces of maleimide and regioselectively direct the nucleophilic attack on one of the two accessible vinylic carbon atoms. For this purpose, we assumed that the stereoselective vinylogous Michael addition between 3-substituted cyclohexenones **12** and *N*-(2-*tert*-butylphenyl)maleimides **9** could be promoted using a primary amine through formation of a chiral dienamine. We screened different primary amines paired with two equivalents of acid cocatalyst to determine the best combination. Accordingly, we found that 9-amino-(9-deoxy)-*epi*-quinine **13** in combination with *N*-Boc-l-phenylglycine **14** afforded the best catalytic salt that can control simultaneous construction of three stereogenic elements in a highly stereoselective way (Scheme 4).

**Scheme 5:**
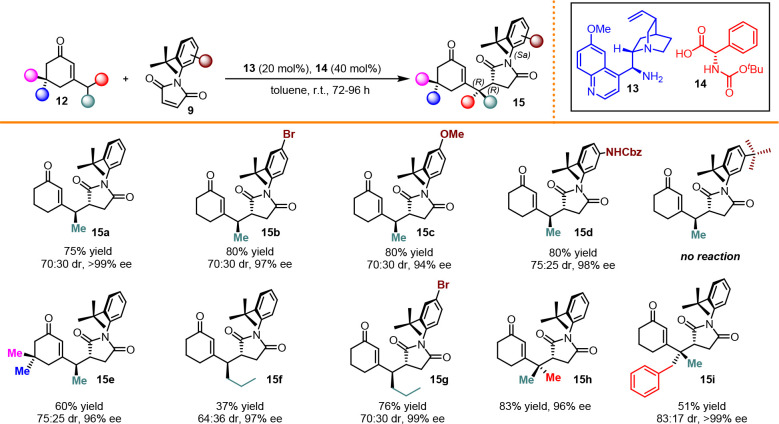
Scope of Desymmetrization

Nuclear Overhauser Effect (NOE) experiments confirmed that the nucleophile always approaches the maleimide through the face, which is not hindered by the *tert*-butyl group, whereas X-rays on the crystals assigned *R*,*R*,*S*_*a*_, i.e., the absolute configuration, to the major diastereoisomer **15a**. Surprisingly, we noted only traces of the product when the acid was not employed; moreover, there seemed to be no correlation between the chirality of the cocatalyst and stereoselectivity of the reaction. The results obtained indicated a good tolerance of the system toward maleimides bearing substituents in 4, but bulky substituents in 5, similar to *tert*-butyl, suppressed the reactivity **15a**–**d** (Scheme 5).

Enones with different substituents on the double bond were less reactive but indicated a good level of conversion when treated with additional electrophilic maleimides **15e**–**g**. Finally, with regard to the γ,γ-disubstituted enones, we recorded two opposite behaviors: non-prochiral γ,γ-disubstituted enones afforded product **15h** with remarkable yields and enantiomeric excesses; however, when the substituents were different, the conversion considerably decreased (**15i**).

**Scheme 6:**
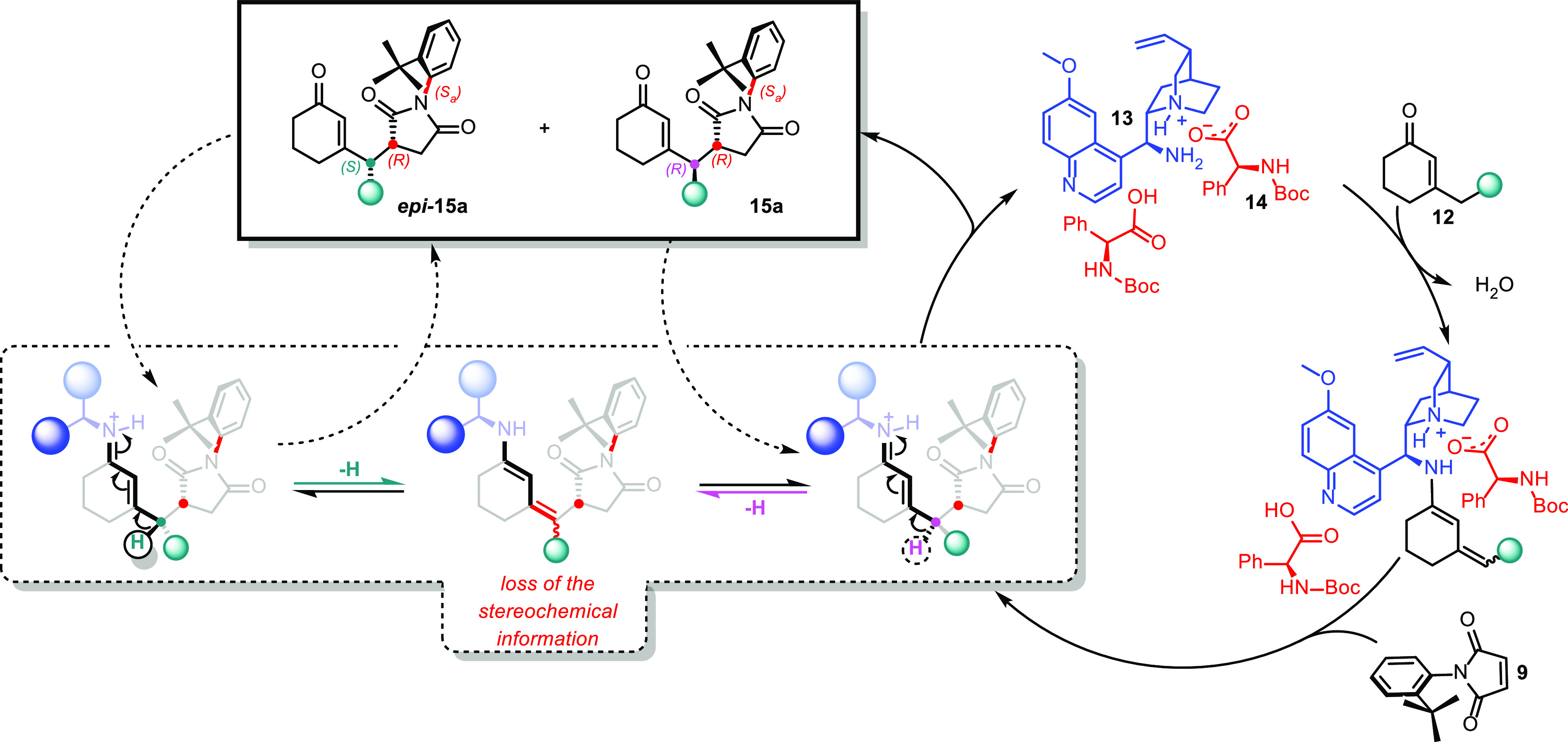
Catalytic Cycle and Erosion of Diasteroselectivity due to Epimerization

Further experiments indicated that the catalyst itself promoted epimerization at the exocyclic stereocenter, which affected the outcome of the diastereomeric ratio. Particularly, **13** could still condense on one of the enantioenriched products **15a**, causing loss of the chiral information of the γ-carbon via dienamine–iminium ion equilibrium (Scheme 6). Therefore, the final diastereomeric ratio (d.r.) observed reflects the thermodynamic stability of two diastereoisomers, i.e., **15a** and *epi***-15a**, and it is not influenced by the catalyst.

**Scheme 7:**
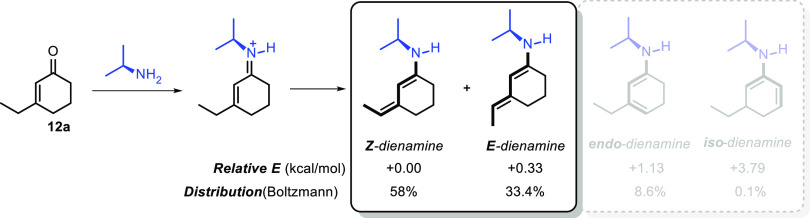
Geometry and Stability of Dienamines

Further, we were interested in elucidating the mechanism of vinylogous Michael addition. Instead of depending only on the experimental results collected so far, to get a better insight into the nature of activation and the stereoselection of the system, we investigated the geometry of the transition state (TS) through DFT computational studies.^16^ We selected the reaction between 3-ethylcyclohex-2-en-1-one **12** and *N*-(2-*tert*-butylphenyl)maleimide **9** as a model, which was catalyzed by 20 mol % of **13** and 40 mol % of **14** (Scheme 4). Before making any further assumption on the TS, we considered that the catalyst (treated as isopropylamine) could form two reactive dienamines, which possess a different configuration of the double bond (E or Z), and the calculations demonstrated that these two reactive dienamines are both formed during the reaction (Scheme 7).

**Scheme 8:**
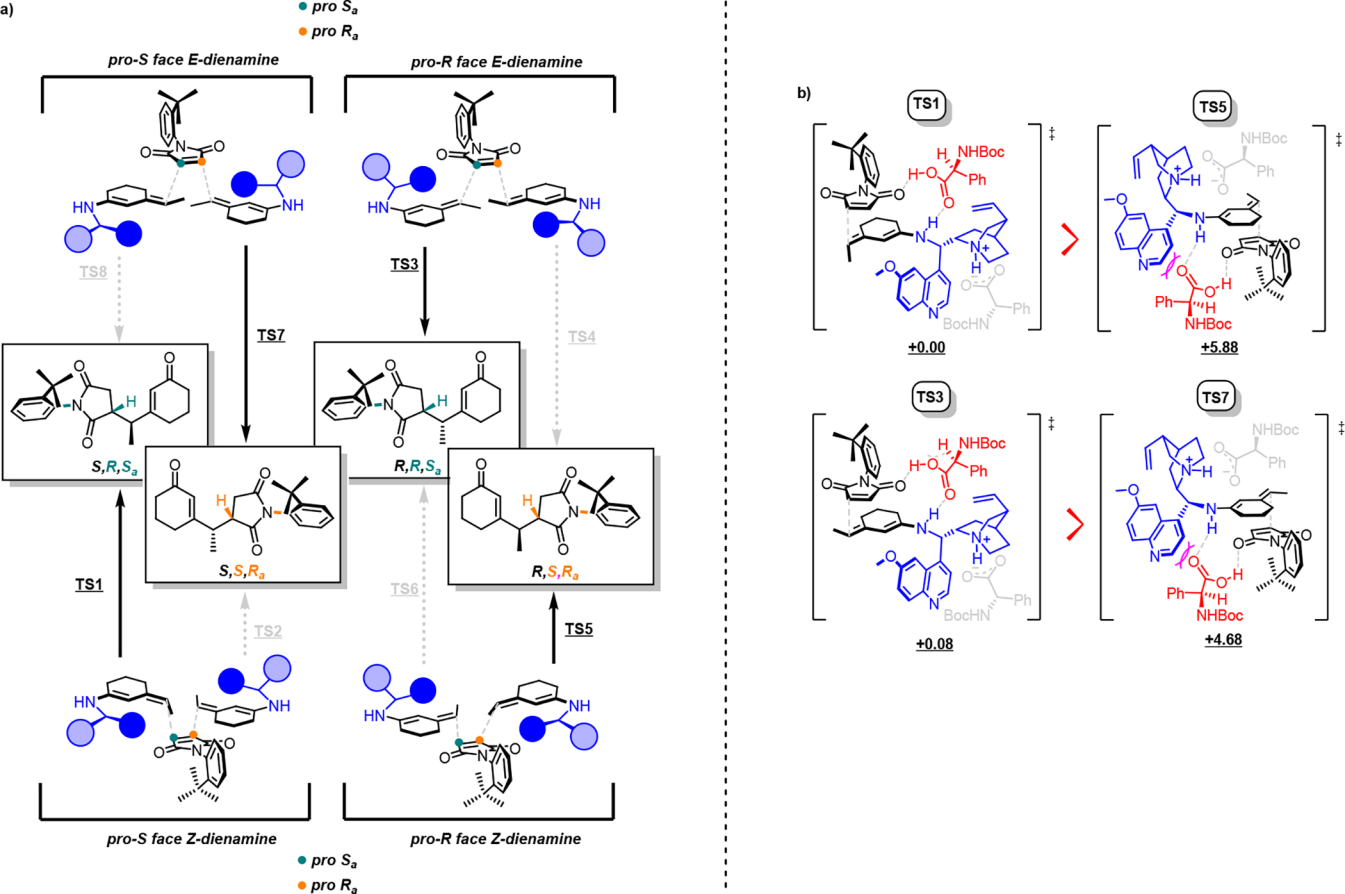
(a) Four Possible Approaches between Maleimide and Each Dienamine Isomer and (b) Four Transition States (TSs) Leading to Two Diastereoisomers Energies reported in kcal/mol.

In the context of the geometry of the TS, we faced the problem of disposing two molecules of the acid cocatalysts. We supposed that while *N*-Boc-phenylglycine protonated the quinuclidine ring, the other *N*-Boc-phenylglycine could bridge reaction partners, thus cyclically activating the system. This model was proposed for four possible approaches between each dienamine isomer and maleimide, considering the two observed diastereoisomers (Scheme 8a).

**Scheme 9:**
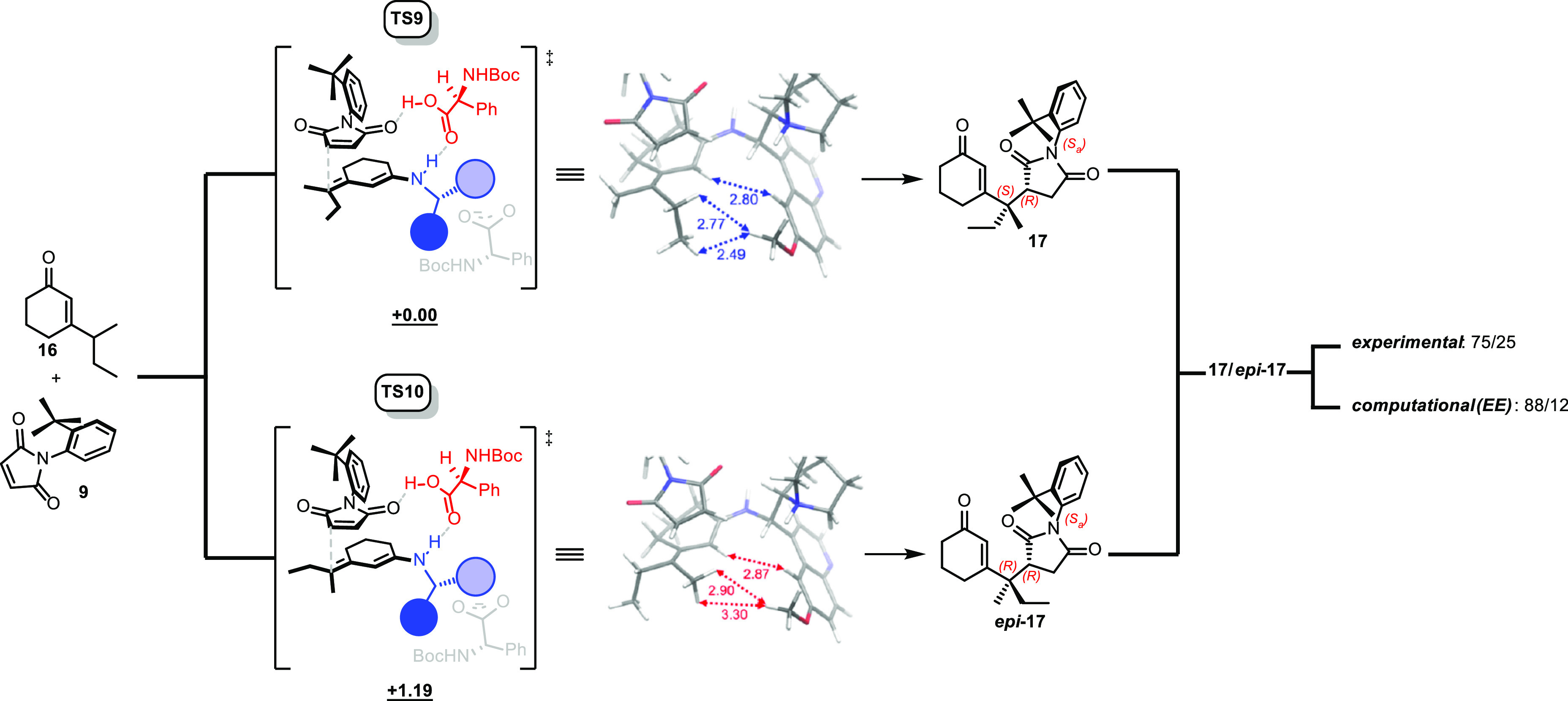
Computed TSs for Nonepimerizable Substrates

Further calculations on intermolecular distances allowed us to exclude half of these TSs. Finally, the remaining four TSs (**TS1**, **TS3**, **TS5**, and **TS7**) were processed using Autodock software, in which each molecule of the cocatalyst was treated as a feasible ligand, which must adjust its position to best fit into the pocket of the rigid receptor formed during these transition states. This ultimate refining calculation allowed us to measure the definitive energies for all the TSs (Scheme 8b). **TS5** and **TS7** were not considered over **TS1** and **TS3** because the quinoline ring pointed toward the cocatalyst and caused more steric clashes that interrupt the bridging interaction. The computational study confirmed the preference of the catalyst to direct Michael addition toward the pro-*S*_*a*_ carbon of maleimide. A prediction of the experimental diastereomeric ratio based on the energy difference between the **TS1** and **TS3** results was incorrect, as the epimerization process covered the effective diastereoisomeric ratio resulting from the simple nucleophilic attack. When the same computational approach was repeated with a nonepimerizable substrate (**16**), the calculated dr agreed well with the experimental results (Scheme 9).

**Scheme 10:**
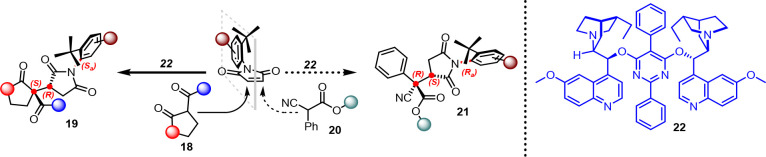
Desymmetrization of *N*-Arylmaleimide through Attack of 1,3-Diketones, β-Ketolactone, and Cyanoesters

Thereafter, we speculated whether a notable level of stereoselection in desymmetrization of *N*-(2-*tert*-butylphenyl) maleimides could be achieved with other types of nucleophiles. Inspired by the study conducted by Melchiorre et al.,^17^ we explored the reactivity of 1,3-dicarbonyls and cyanoesters using pyrimidine-bridged cinchona alkaloid catalyst **22** (Scheme 10).^18^

**Scheme 11:**
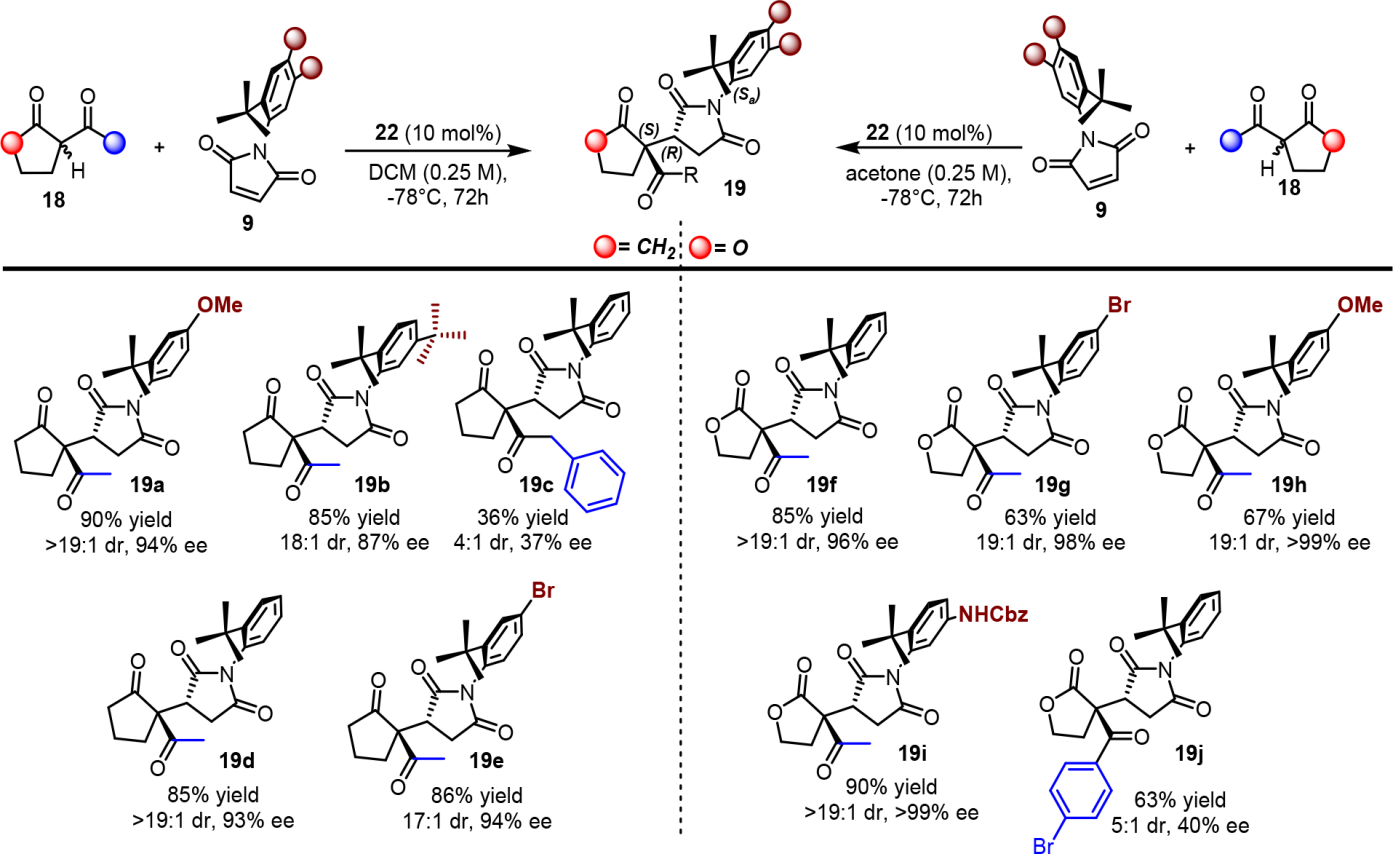
Scope of Michael Addition of 1,3-Dicarbonyls to Maleimides

We found that even in this case, the cinchona alkaloids represented a valid tool in recognizing the atropotopic faces of pro-axially chiral maleimides. In the presence of 1,3-diketones and β-ketolactones, a high level of conversion and stereoselection were achieved using **22**, which directed the *Si* face of the nucleophile toward the pro-*S*_*a*_ carbon of the maleimide from its accessible face, thus providing a single diastereoisomer with the *S,R,S*_*a*_ absolute configuration (Scheme 11). This system tolerated electron-poor and electron-rich maleimides as well as those bearing a highly sterically demanding *tert*-butyl group in position 5 of the phenyl group. In most cases, the catalyst controlled the concomitant formation of three stereogenic elements except in some cases, in which there was a critical decrease in the stereocontrol when aromatic rings were present on the nucleophile (**19a**–**j**).

**Scheme 12:**
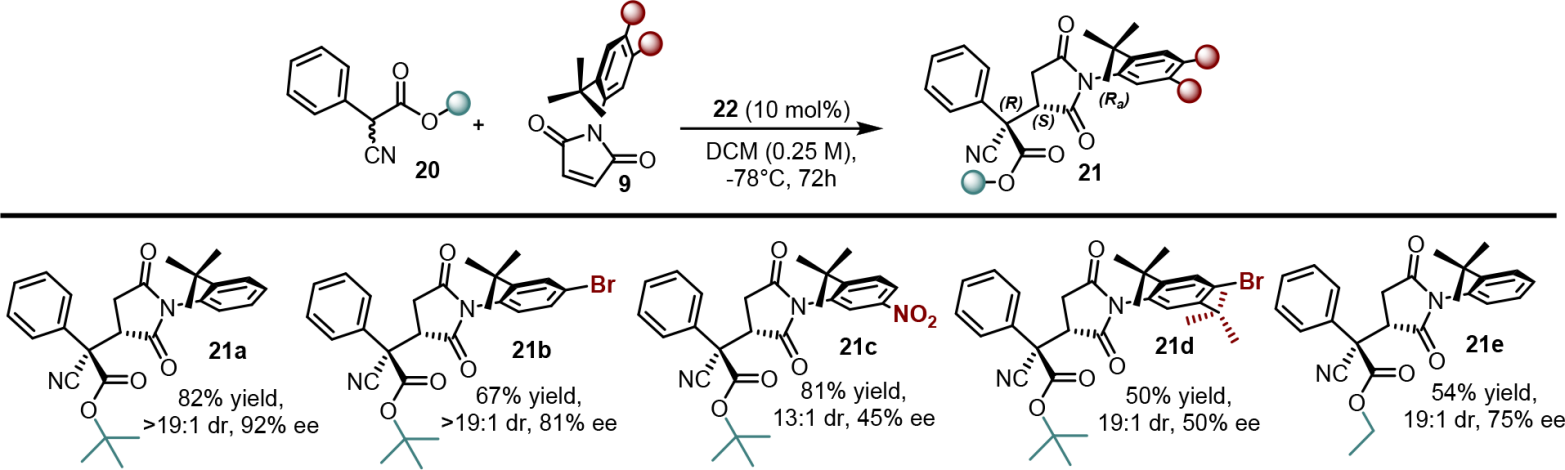
Scope of the Reaction for α-Cyanoesters

Moreover, we explored the behavior of **22** in promoting Michael addition of α-cyano esters, again underlining the high adaptability of catalyst **22** toward different nucleophiles (Scheme 12). Indeed, **22** still ensures good enantiocontrol but with reactants bearing small substituents. The final product was obtained with the *R,S,R*_*a*_ configuration, which is the opposite with respect to that observed for 1,3-dicarbonyls (**21a**–**e**).

**Figure 1:**
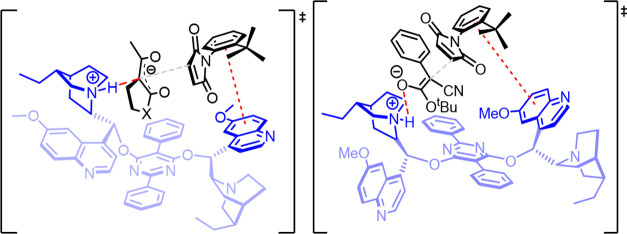
Transition states (TSs) for the two types of nucleophiles.

In the context of the reaction mechanism, we hypothesized that the stereochemical outcomes depend on secondary interactions, such as π-stacking, provided by the quinoline group on one side of catalyst **22** and the hydrogen bond provided by the quinuclidine core on the other side. These two units generate a chiral pocket that helps create a rigid closed TS of the reaction. When we used cinchona alkaloids with free OH, a considerably low enantiomeric excess was obtained, confirming the fundamental role of the dimeric nature of **22** to reach high stereocontrol. Therefore, we envisaged a TS, in which the catalyst induces π-stacking interaction between the quinoline and aromatic ring of the maleimide, thus exposing the *R*_*a*_ prochiral carbon of maleimide to the nucleophile activated by the quinuclidine ring (Figure 1). This could be the reason for a decrease in stereoselection caused by using aromatic substituted 1,3-diketones or β-ketolactones: the aromatic moiety can interfere with π-stacking between the maleimide and quinoline moiety of the catalyst.

**Scheme 13:**
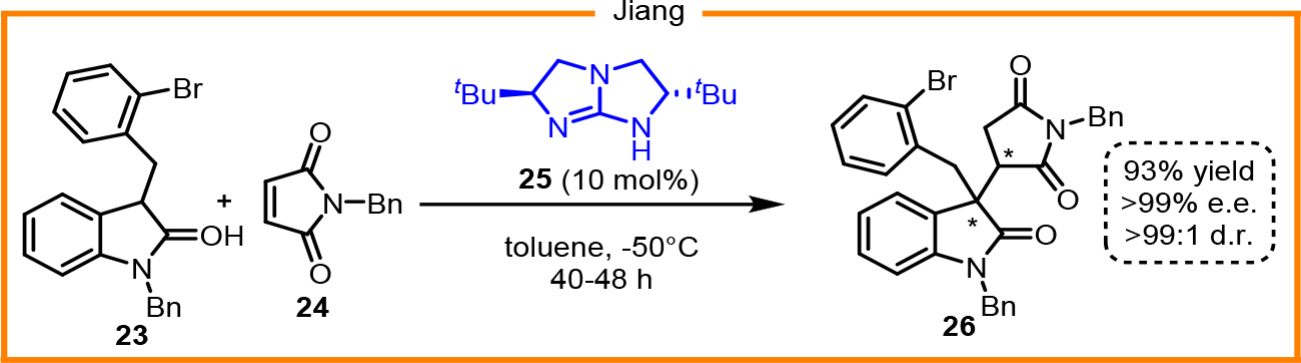
Asymmetric Michael Addition of 3-Substituted Oxindoles to Arylmaleimides

Following the atropselective organocatalytic Michael additions to maleimides, we considered oxindoles as possible nucleophiles, as they can afford chiral structures pivotal for pharmaceutical applications.^19^ In 2012, Jiang studied asymmetric Michael addition between oxindoles and arylmaleimides (Scheme 13),^20^ and along with this study, several other cases had been reported; however, atropselective variants are yet to be explored.

**Scheme 14:**
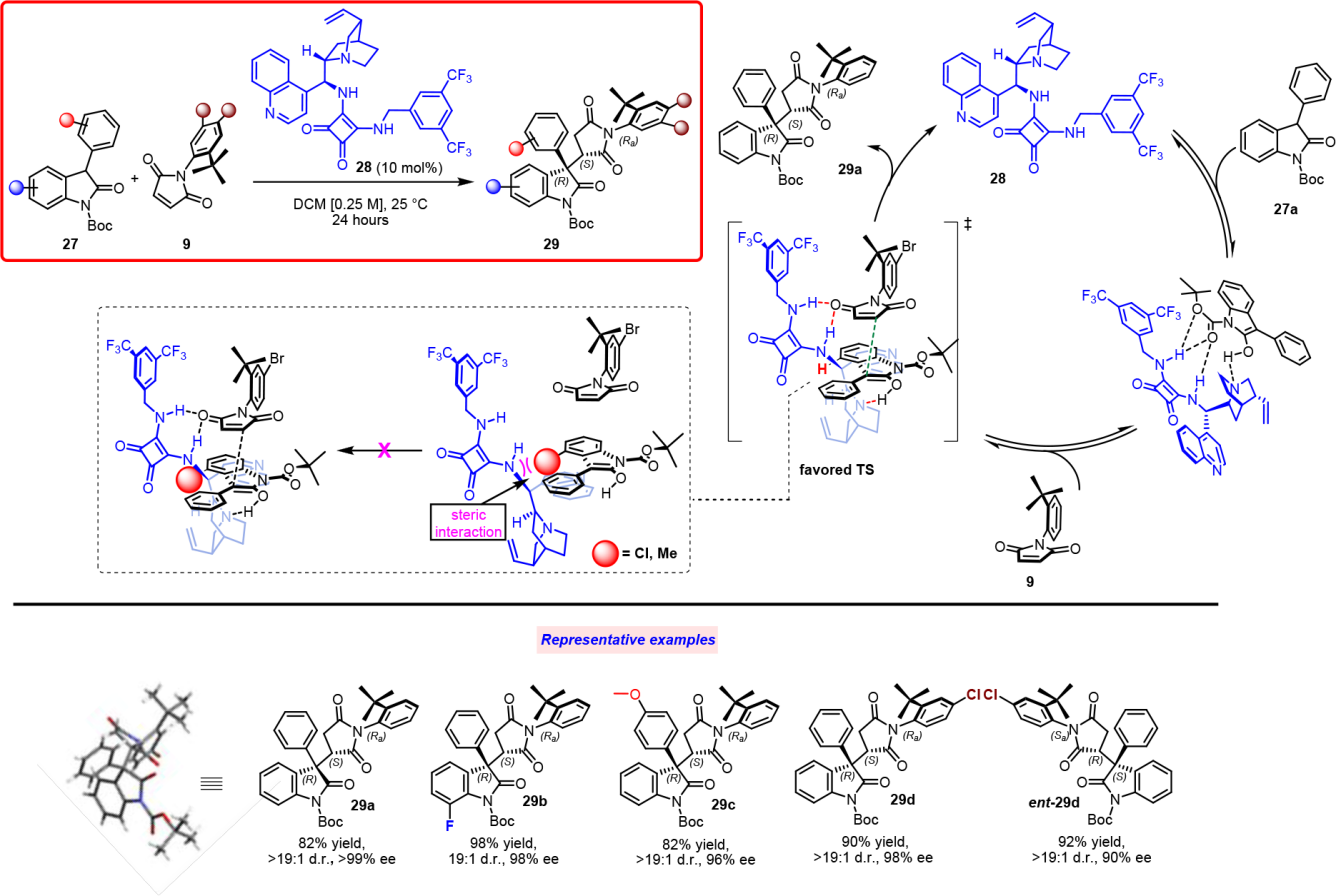
Atropselective Michael Addition of Oxindoles to *N*-(2-*tert*-Butylphenyl)maleimides via Organocatalytic Desymmetrization

Therefore, we examined the reaction between *tert*-butyl-2-oxo-phenylindoline-1-carboxylates **27** and *N*-(2-*tert*-butylphenyl)maleimides **9** (Scheme 14).^21^ The approach was to exploit the ability of a functionalized cinchona alkaloid catalyst to transfer defined stereoselectivity in forming the stereogenic axis via addition of oxindole to *N*-(2-*tert*-butylphenyl)maleimide.

The first catalyst investigation showed that thiourea-functionalized cinchona alkaloid can exhibit promising results in terms of yield and stereoselectivity, thereby directing the attention toward bifunctional catalysts. The squaramide-functionalized 9-amino-(9-deoxy)-*epi*-cinchonidine **28** turned out to be the best selection for activating oxindole through deprotonation and maleimide using the coordinating squaramidic group. Subsequently, the product was obtained as a single diastereoisomer with excellent yield and stereocontrol on three stereogenic elements (**29a**–**d**). Notably, the same level of stereoselectivity was obtained employing the pseudoenantiomer of catalyst **28** (*ent*-**29d**). The absolute configuration (*R,S,R*_*a*_) was determined via X-ray analysis and suggested that the catalyst associates with the maleimide carbonyl group from the *Si* atropotopic side, remotely controlling the attack of oxindole toward the *Re* face of the C_b_ of the double bond. The reaction scope demonstrated good tolerance for meta- and para-substituents on the maleimide phenyl ring. Oxindoles with different aromatic substituents at C3 were effective in the reaction unless an excessively bulky group was introduced. Finally, monosubstituted oxindole aromatic cores were tolerated, whereas disubstituted oxindole aromatic cores afforded lower yields. Oxindoles with substituents at C4 did not react at all. It is reasonable that catalyst **28** approaches the nucleophile close to the hydrogen at C4, with the squaramide moiety directed outside the oxindole plane; hence, when substituents were present at C4, strong repulsion occurred, suppressing the reaction. To gain additional insights into the catalytic mode of action, the reaction was executed with a small catalyst such as diazabicyclo[2.2.2]octane (DABCO). In this case, complete conversion was obtained in 24 h, although with a low dr (4:1). This implies that nucleophile activation is controlled by the tertiary nitrogen, whereas the squaramide with concomitant activation and coordination on maleimide helps control the geometry of the approach, thereby creating a compact TS and transferring the chiral information to the product.

**Scheme 15:**
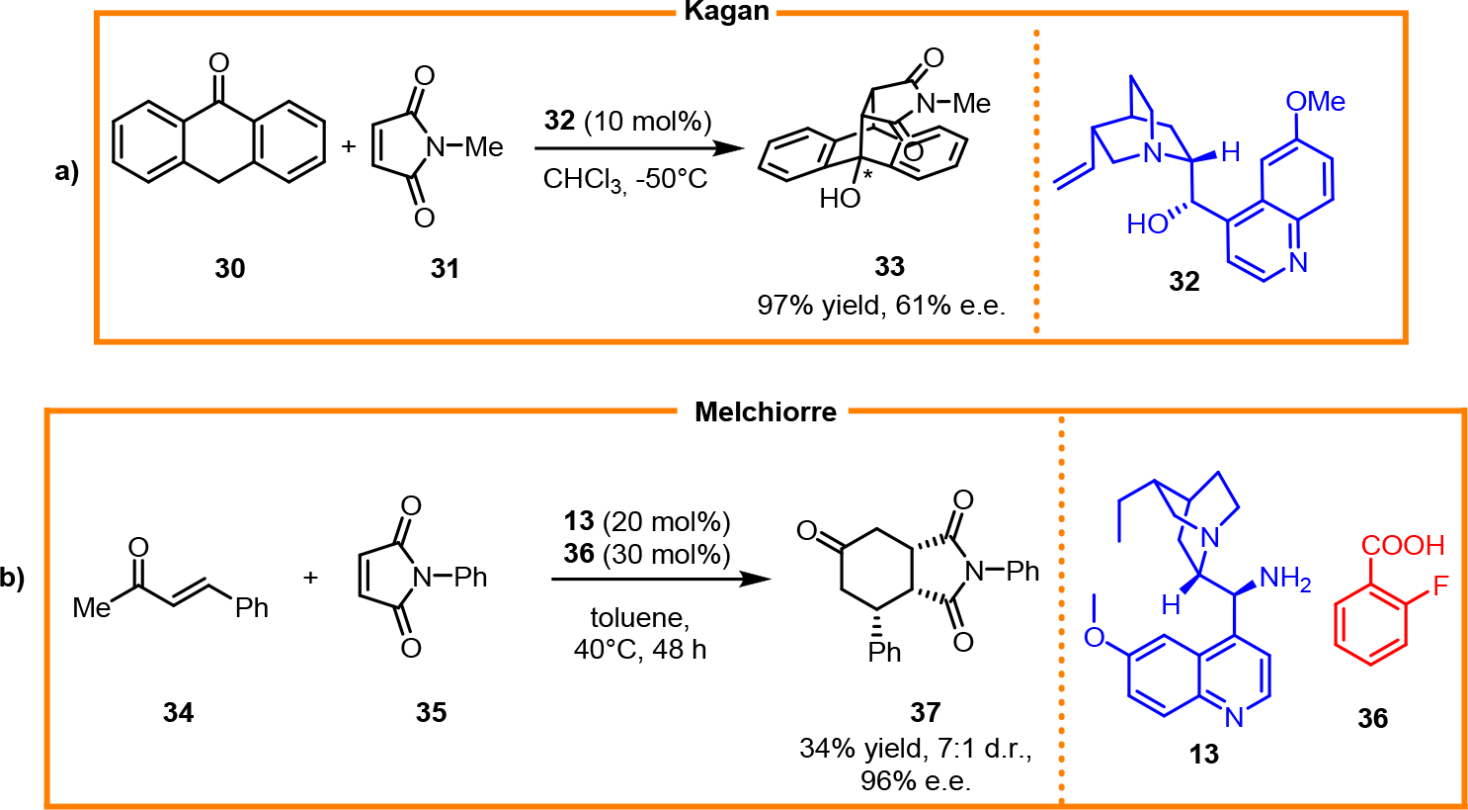
(a) First Base-Catalyzed Asymmetric Diels–Alder Reaction and (b) Organocascade Reaction of Enones Catalyzed by a Primary Amine

Encouraged by the excellent results obtained from desymmetrization of maleimides, we decided to explore this concept via the Diels–Alder (DA) reaction.^22^ The first base-catalyzed asymmetric DA reaction was reported by Kagan,^23^ in which *N*-methylmaleimide reacted with anthrone in the presence of a catalytic amount of quinidine to afford the optically active product with excellent yields (Scheme 15a). The primary amine-catalyzed organocascade reaction between enones and maleimides was reported in 2009 by Melchiorre,^24^ through which formal DA cycloadducts were obtained with great enantioselectivity (Scheme 15b).

**Scheme 16:**
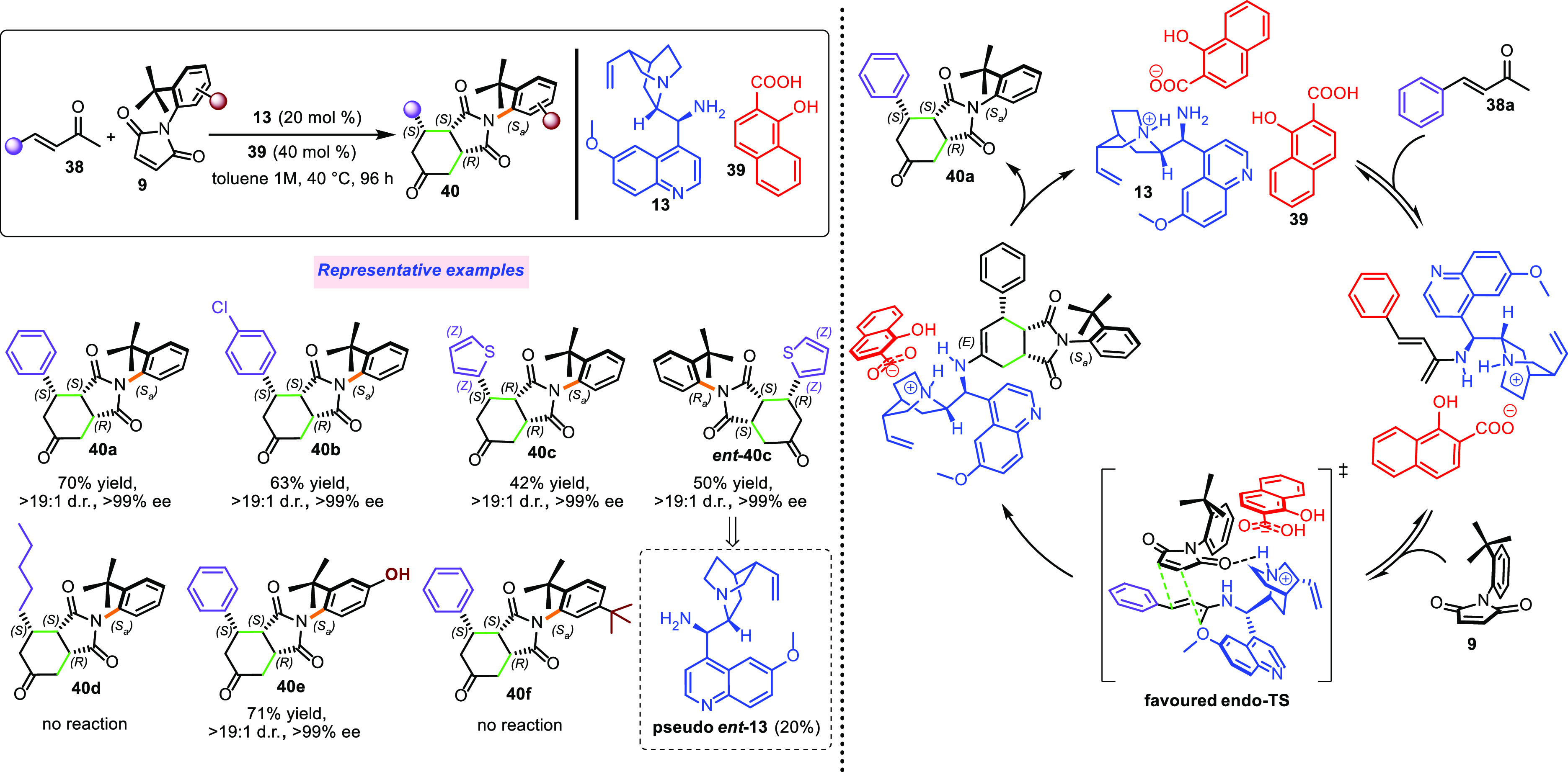
Atropselective Diels–Alder Desymmetrization of *N*-Arylmaleimides

Therefore, we envisioned whether an atropselective version can be achieved under the influence of a primary amine organocatalyst, and in 2015, we realized the formal DA cycloaddition of α,β-unsaturated enamines to *N*-(2-*tert*-butylphenyl)maleimide, through which four stereogenic elements were simultaneously and selectively formed (Scheme 16).^25^

The intention was to use a chiral primary amine catalyst to activate α,β-unsaturated ketones and form a stereodefined enamine intermediate that will serve as diene in atroposelective cycloaddition. Amino quinine **13** was revealed to be the best catalyst in combination with two equivalents of 2-hydroxybenzoic acid **39**, forming the product **40** as a single diastereoisomer with high yield and stereocontrol. The reaction proceeds based on the endo rule during the cycloaddition step, as typical for dienophiles possessing suitable conjugating substituents. The catalyst drives the diene approach on the opposite side from the *tert*-butyl group and interacts with the atropotopic *Si* face of maleimide, suggesting a favored TS in which stabilizing hydrogen bonding occurs between the carbonyl of the electrophile and quaternary nitrogen of the quinuclidine. Again, 9-amino-(9-deoxy)-*epi*-quinine **13** could remotely control the configuration of the stereogenic axis and that of three contiguous stereocenters. Various unsaturated ketones bearing different substituents on the aromatic ring reacted smoothly; substituents in position 4 of the maleimide were tolerated, whereas those in position 5 sterically hampered the approach of enamine (**40a**–**f**). Catalyst **13** could not realize the DA reaction when alkyl substituents were used, and this acted as a limitation to the reaction scope. Generally, for cinchona organocatalysts, access to both enantiomers of a product is easily realized. Accordingly, herein, the pseudo-enantiomer *ent***-13** catalyst produced *ent***-40c** with high stereoselectivity.

## 3 Synthesis of Alkylidene Cyclohexanones via Axially Enantioselective Knoevenagel Condensation

**Scheme 17:**
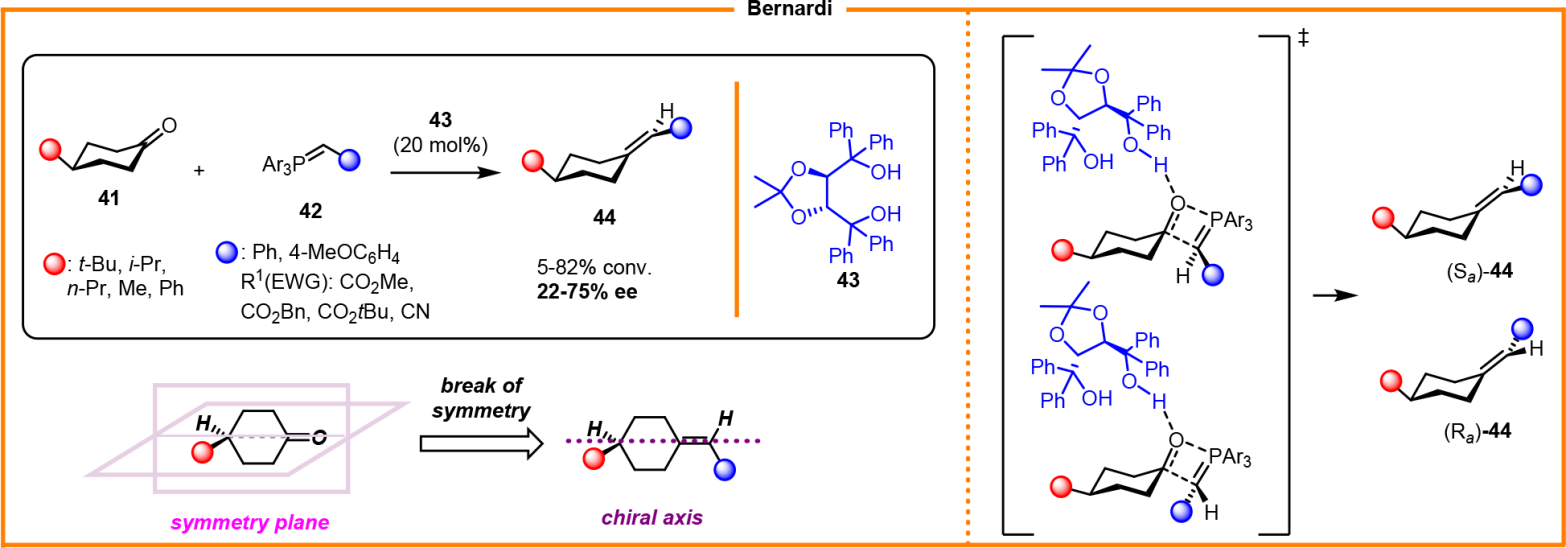
First Asymmetric Wittig Reaction for the Synthesis of Alkylidene Cyclohexanes

In addition to atropisomerism deriving from a restricted rotation around a single bond, axially chiral compounds include spiranes, allenes, and alkylidenecycloalkanes.^26^ Although asymmetric organocatalytic syntheses have been explored for the first two types,^27^ the enantioselective synthesis of alkylidenecycloalkanes is underdeveloped. Bernardi reported the first asymmetric synthesis of axially chiral alkylidenes via an organocatalytic Wittig reaction (Scheme 17).^28^

The [2 + 2] cycloaddition step between 4-substituted cyclohexanones **41** and stabilized phosphorus ylides **42** was catalyzed by TADDOL **43**, which was able to break the symmetry plane of the substrate, thereby affording the final enantioenriched product **44** with low enantiocontrol.

With the aim of realizing enantioselective synthesis of axially chiral alkylidene scaffolds, we decided to exploit an asymmetric Knoevenagel olefination reaction.^29^ The first asymmetric Knoevenagel condensation was realized by List,^30^ who conducted the reaction between α-branched aldehydes and 1,3-dicarbonyl derivatives. Under the effective control of a cinchona primary amine catalyst, enantioenriched olefins were obtained through a dynamic kinetic resolution pathway.

**Scheme 18:**
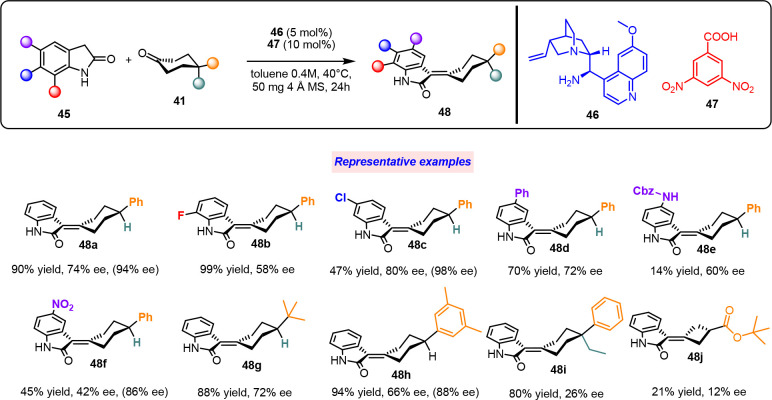
Axially Enantioselective Knoevenagel Condensation

To realize axially chiral-selective Knoevenagel condensation,^2^ we explored the reaction between 4-substituted cyclohexanones **41** and oxindoles **45** under the control of 9-amino-(9-deoxy)-*epi*-quinidine **46** as the catalyst and 3,5-dinitro benzoic acid **47** as the cocatalyst (Scheme 18).

**Scheme 19:**
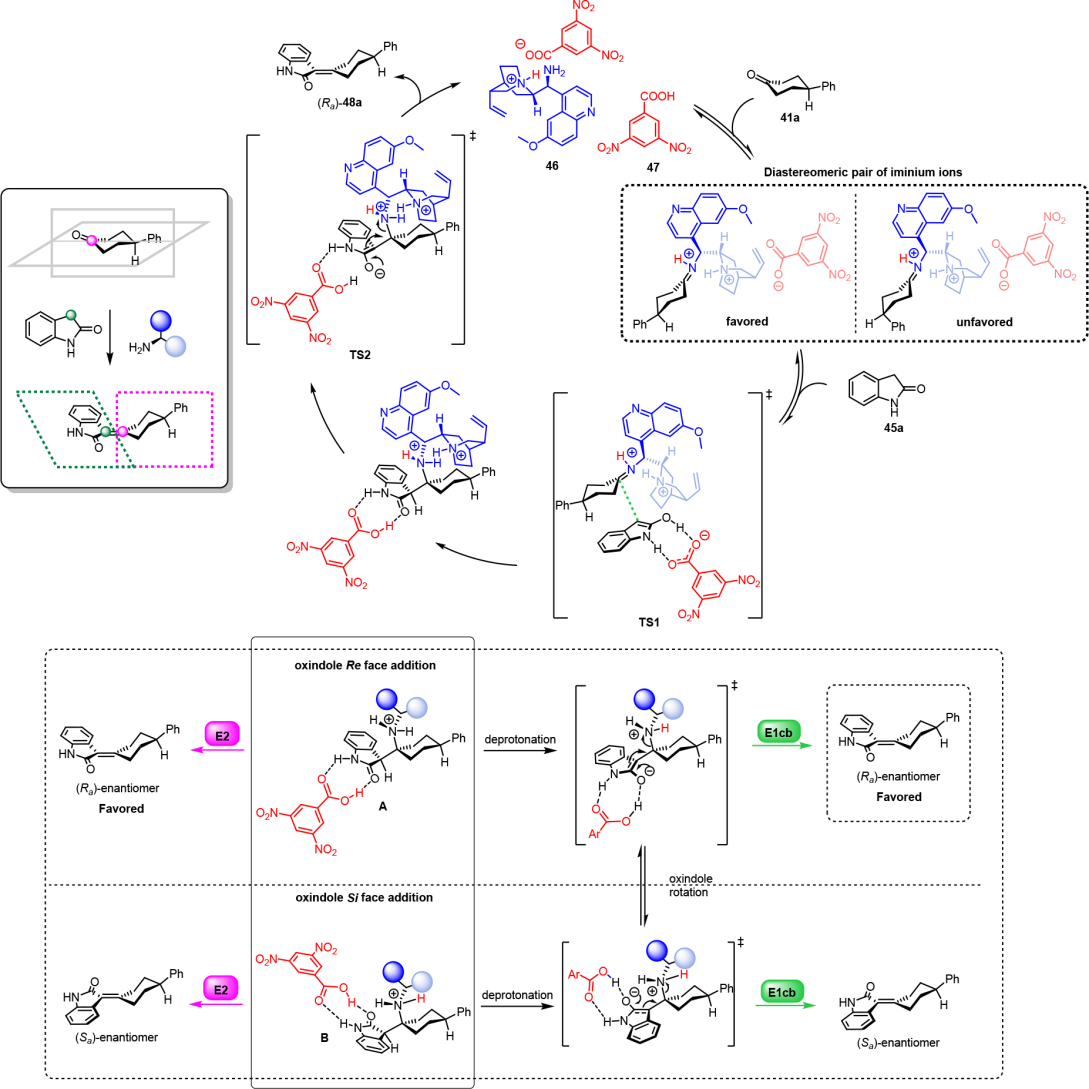
Proposed Mechanism for Enantioselective Knoevenagel Condensation

Differently substituted oxindoles were tested, and important results were obtained. Highly insoluble substrates and oxindole bearing a strong electron-withdrawing group resulted in a decrease in yield and stereoselectivity (**48c**–**f**). Cyclohexanones with aromatic and aliphatic substituents produced excellent yield and demonstrated good-to-moderate enantiocontrol (**48g**–**i**). Interestingly, a cyclobutanone derivative could be used; however, the corresponding olefination product could be obtained in moderate yield and enantioselectivity (**48j**). A further enantioenrichment of the product was observed when the crude reaction mixture was filtered using a PTFE syringe filter. This outcome was caused by preferential precipitation of scalemic fractions of the products, thereby leaving the enriched major enantiomer in solution (ee values in brackets for **48a**, **48c**, **48f**, **48h**). The absolute configuration was assigned based on the TD-DFT calculations of the electronic circular dichroism spectra and set as *R*_*a*_. To elucidate aspects of the reaction mechanism, DFT calculations were performed in detail (Scheme 19).

The search of the TS resulted in locating two lowest energy structures corresponding to the selective attack of the *Re* face of the iminium ion from both faces of the oxindole (intermediates **A** and **B**, Scheme 19). Two possible elimination paths were considered: E2 and E1cb. The first path is stereospecific, and the final stereochemistry would depend on the geometry of the addition of TS1. The second path is stereodetermining, as two interchangeable diastereomeric rotamers of the enolate are formed (TS2) under the Curtin–Hammett profile. The ratio of the products depends on the energy difference between the E1cb TSs and is solely determined by the catalyst. All the corresponding E2/E1cb TSs were located at certain energy values, indicating that the *R*_*a*_ product was preferred in both elimination routes. For Knoevenagel condensation, the E1cb path was considered, as it possessed low energy, and the calculated product ratio agreed well with the experimental value. In this case, the catalyst played a fundamental role in controlling the stereochemistry of the final product.

## 4 Direct Synthesis of Conformationally Restricted Diastereoisomers

**Scheme 20:**
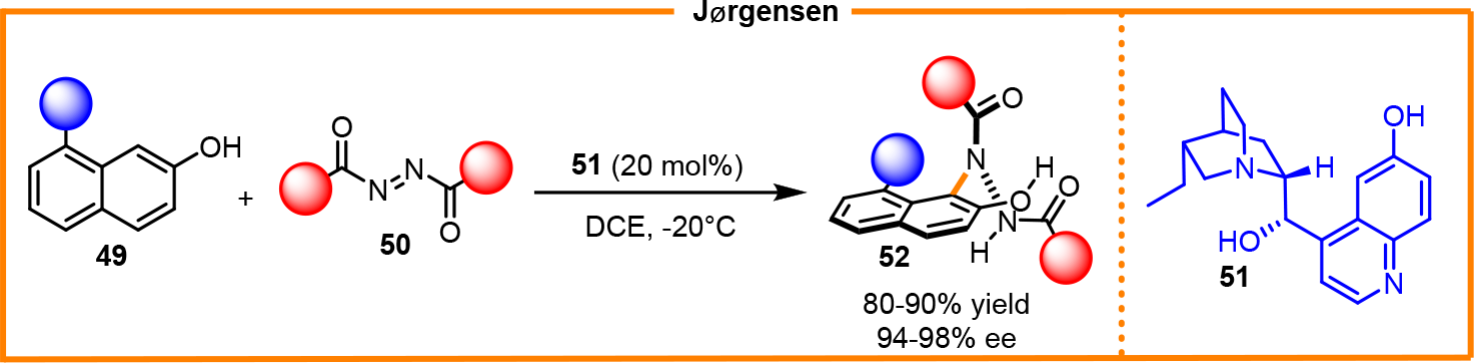
Atropselective Amination Reaction of Naphthols

Direct synthesis of C–C and C–heteroatom atropisomers has been a challenge, which is addressed by metal-catalyzed reactions. The use of coupling reactions provides the preferential route to synthesize atropisomeric compounds, particularly biphenyl and binaphthyl.^31^ Over 15 years, organocatalysis has been able to face the aforementioned challenge, introducing an alternative method to classical desymmetrization or kinetic or dynamic kinetic resolution, which has been successfully applied to synthesize atropisomers. A venerable case has been realized by Jørgensen, who reported formation of β-hydrazino-naphthol atropisomers through the reaction between azodicarboxylates and β-naphthol catalyzed by a chiral organic base **51**. This case can be considered as one of the first organocatalytic enantioselective direct syntheses of C–N atropisomer **52** (Scheme 20).^32^

**Scheme 21:**
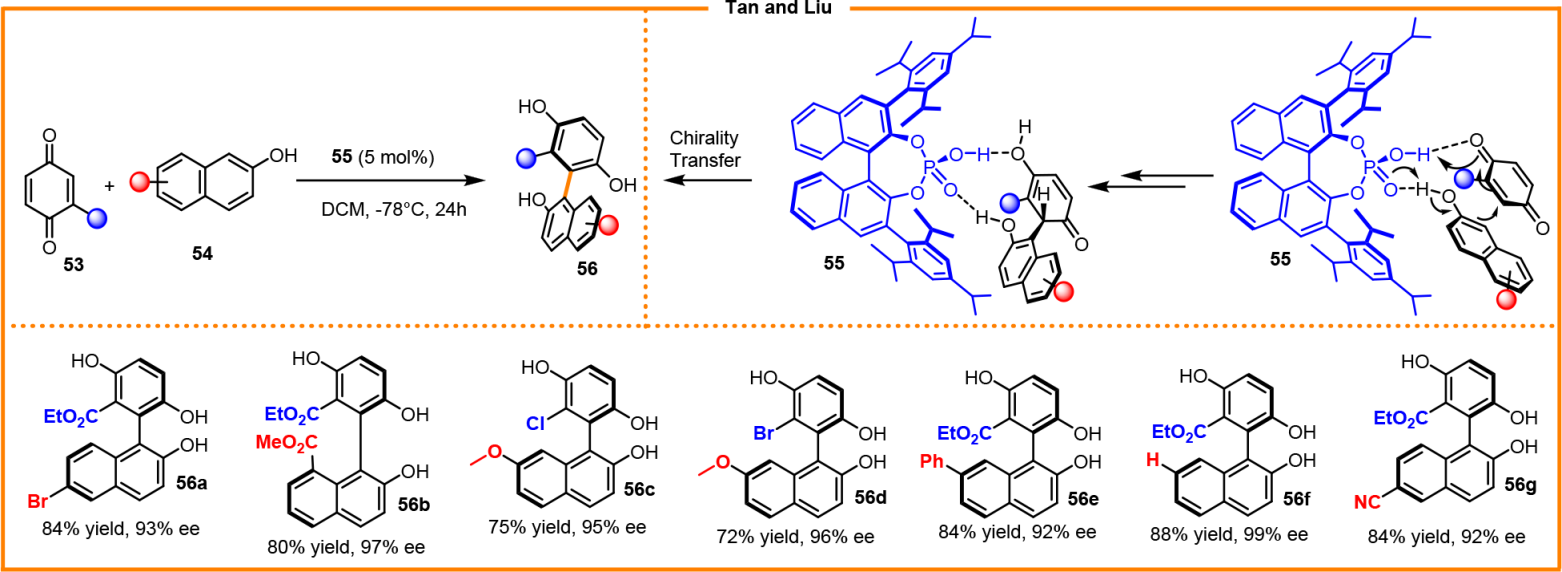
Brønsted Acid Catalyzed Atropselective Synthesis of Biaryldiols

However, a step forward has been achieved when C–C atropisomers have been prepared via direct arylation of quinones. In 2015, Tan and Liu reported the enantioselective synthesis of biaryldiols catalyzed by chiral phosphoric acid, such as (*S*)-TRIP **65** (Scheme 21).^33^

**Figure 2:**
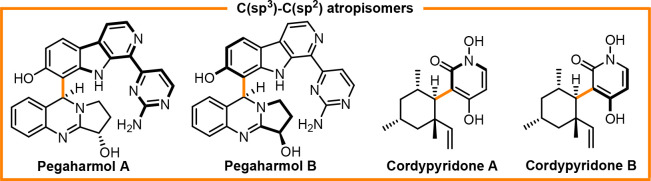
Examples of natural products with a C(sp^2^)–C(sp^3^) stereogenic axis.

Excellent reactivity was achieved via catalyst-promoted conjugate addition of naphthols to quinones followed by aromatization of the resulting intermediate. The high enantioselectivity observed in the final biaryldiols **56a**–**g** was achieved under the influence of the H-bonding network that assists the addition and aromatization steps. Other studies have highlighted the efficiency of different types of organocatalysts to realize analogous direct coupling reactions.^34^ In addition to the studies conducted by Bella and Miller using a cinchona alkaloid and a short peptide as the catalyst, respectively,^35^ the synthesis of C(sp^2^)–C(sp^3^) atropisomers herein represents another example of direct synthesis of C–C atropisomers. This field, which has been rarely explored, is an interesting and challenging topic that draws inspiration from natural substances with complicated molecular architectures, such as cordypyridone A and B and pegaharmol A and B (Figure 2).

**Figure 3:**
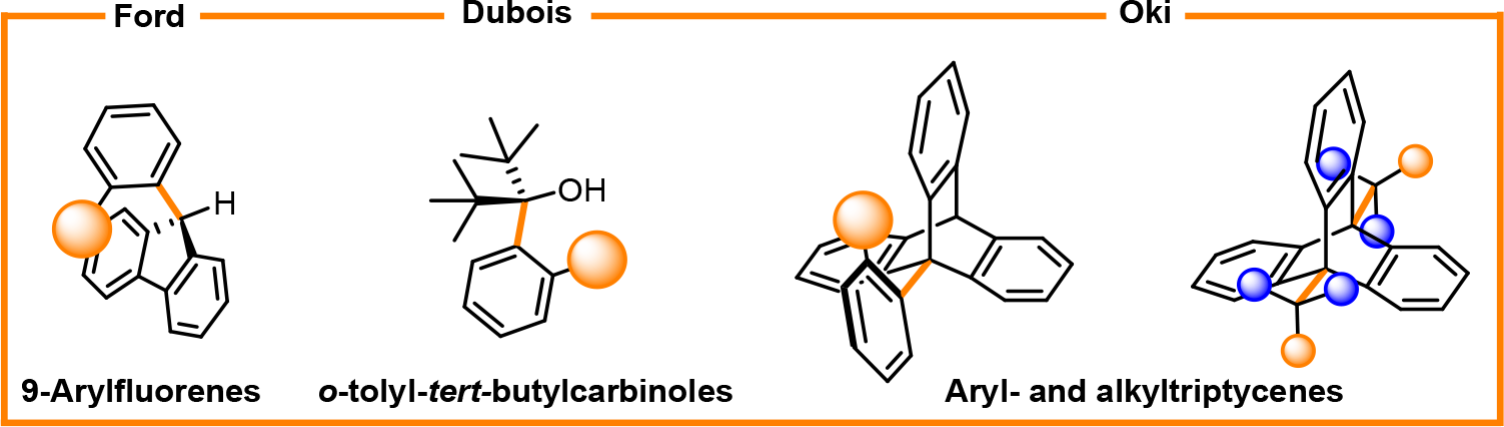
Examples of C(sp^2^)–C(sp^3^) atropisomers.

Furthermore, C(sp^2^)–C(sp^3^) conformational diastereoisomeric atropisomers were developed in the second half of the 1970s, when O̅ki and Ford reported the isolation and rotational barrier determination of aryltriptycenes and 9-aryfluorenes, respectively. Dubois observed conformational isomerism in *o*-tolylcarbinols (Figure 3).^36^

**Scheme 22:**
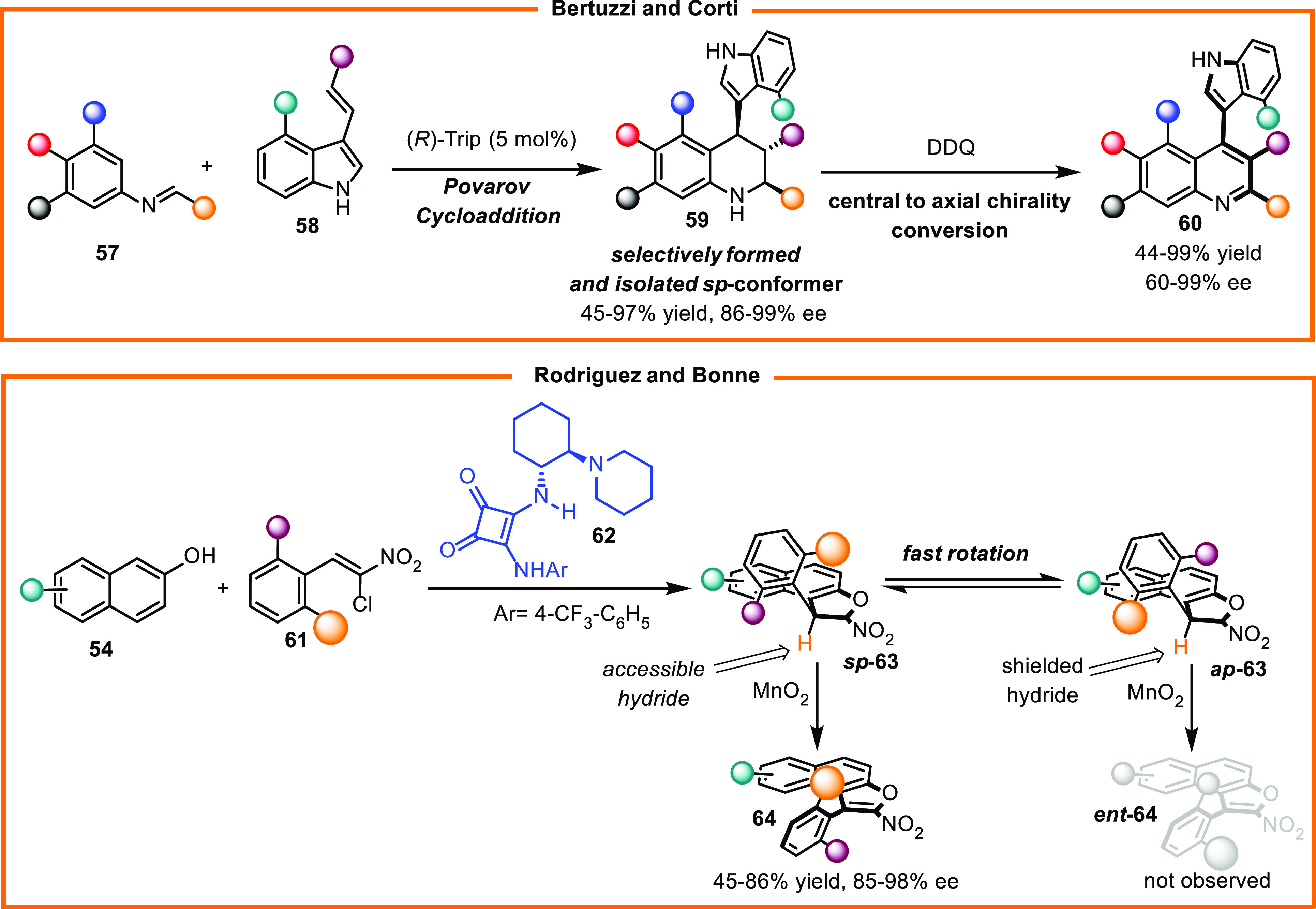
Synthesis of Biaryl Atropisomers via Chirality Conversion of C(sp^2^)–C(sp^3^) Conformational Diastereoisomers

Conformational diastereoisomers featuring a relatively slow barrier to rotation have been observed in sporadic cases and are considered effective intermediates for realizing synthesis of biaryl atropisomers through central-to-axial chirality conversion, as highlighted by studies conducted by Bertuzzi and Corti and Rodriguez and Bonne (Scheme 22).^37^

**Scheme 23:**
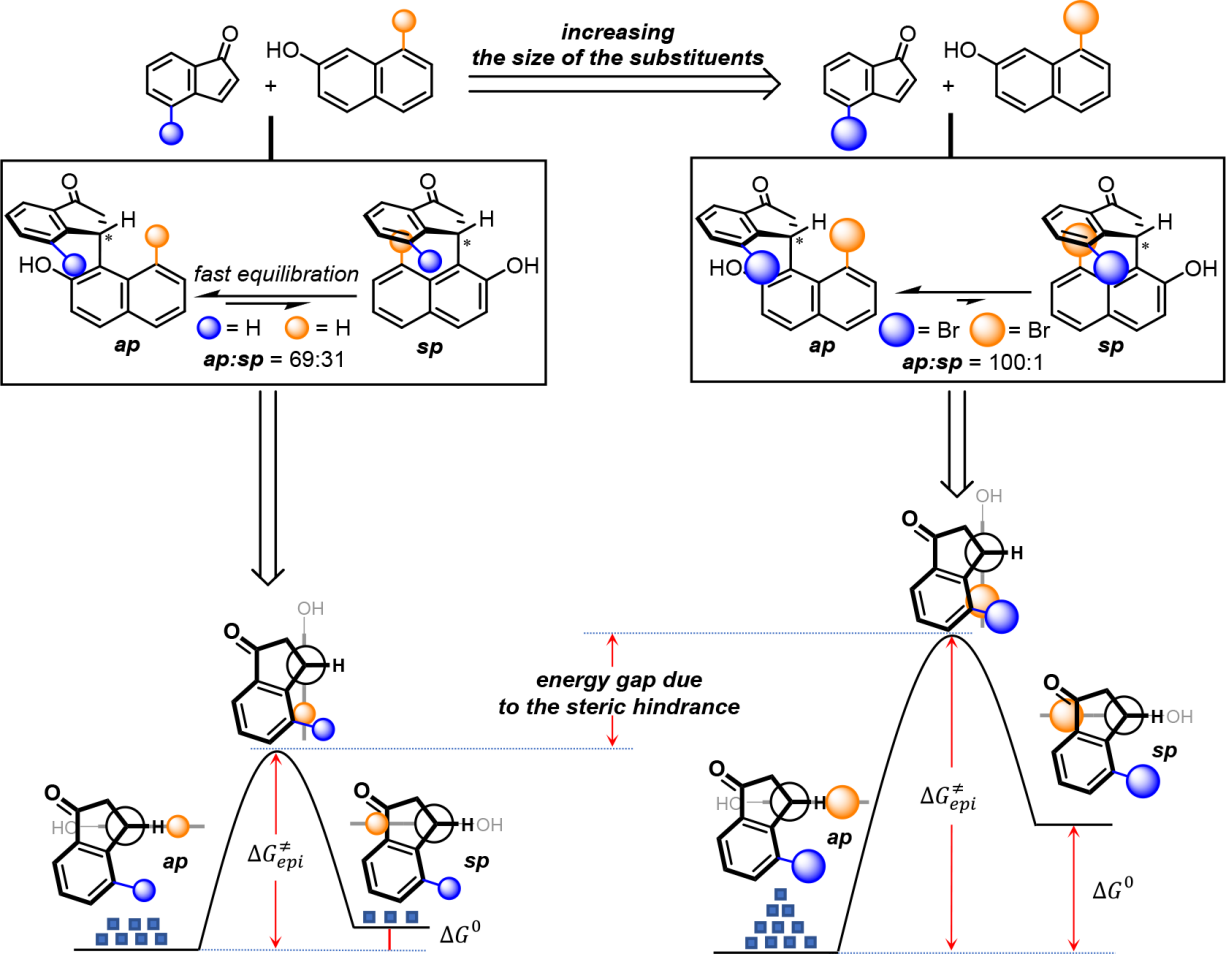
Thermodynamic Control on the Stereogenic Axis Conformation Owing to Steric Hindrance

We explored enantioselective preparation of C(sp^2^)–C(sp^3^) conformational diastereoisomers, achieving thermodynamic control over the axial conformation of indanone derivatives by reacting substituted β-naphthols and inden-1-ones via iminium ion catalysis.^38^ We established that β-naphthol and inden-1-one efficiently reacted and generated an enantioenriched mixture of chiral antiperiplanar (*ap*) and synperiplanar (*sp*) conformers, which were evident at the NMR spectroscopy time scale and room temperature (Scheme 23).^39^

**Scheme 24:**
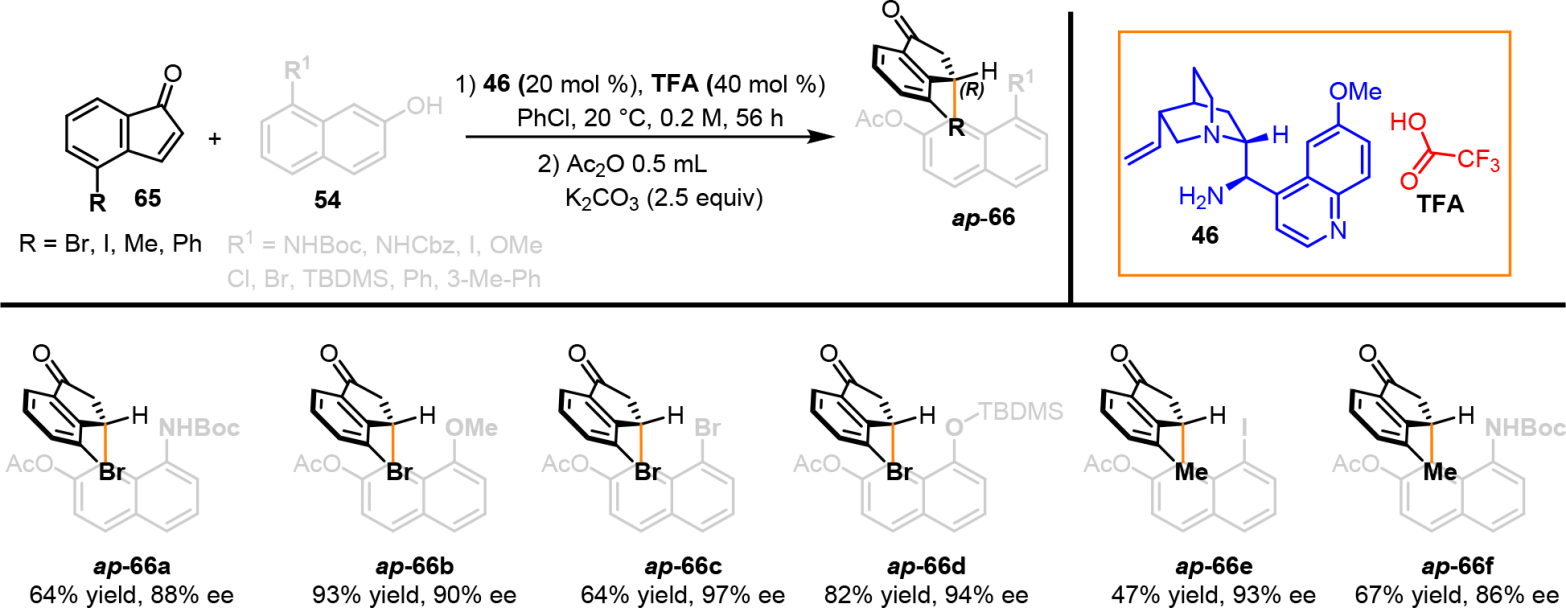
Controlling the Axial Conformation via Organocatalyzed Friedel–Craft Alkylation

This observation led us to confirm that a slow rotation along the formed single bond was responsible for the conformational equilibrium observed. We effectively measured a rotational energy barrier of 17.8 kcal/mol considering the steric interaction between C4 of indanone and C8 of β-naphthols responsible for the relatively high rotational energy barrier. We studied this effect in detail, and by increasing the steric hindrance at C8 of naphthol and C4 of indanone, we attempted to obtain a thermodynamic preference for one conformer and rotationally stable C(sp^2^)–C(sp^3^) atropisomers. Our concept was successfully addressed by using large substituents at ideal positions. A gradual increment in the rotational energy barrier, accompanied by an increase in the conformational ratio in favor of the *ap* conformer, was observed by placing a bromine atom and NHBoc at C8 of β-naphthol **54** and C4 of indene-1-one **65**, respectively (Scheme 24). The broad applicability of the F–C alkylation was demonstrated by preparing a large number of alkylated indanones with good yield and enantioselectivity (*ap***-66a–f**). The sole *ap* conformer was preferred when large substituents were placed at both positions. This result in agreement with our assumption revealed the difficulty to experimentally determine the rotational energy barrier for the *ap* to *sp* epimerization process, suggesting that the existence of the sole *ap* conformer is the result of thermodynamic control over the conformation of the C(sp^2^)–C(sp^3^) stereogenic axis.

**Scheme 25:**
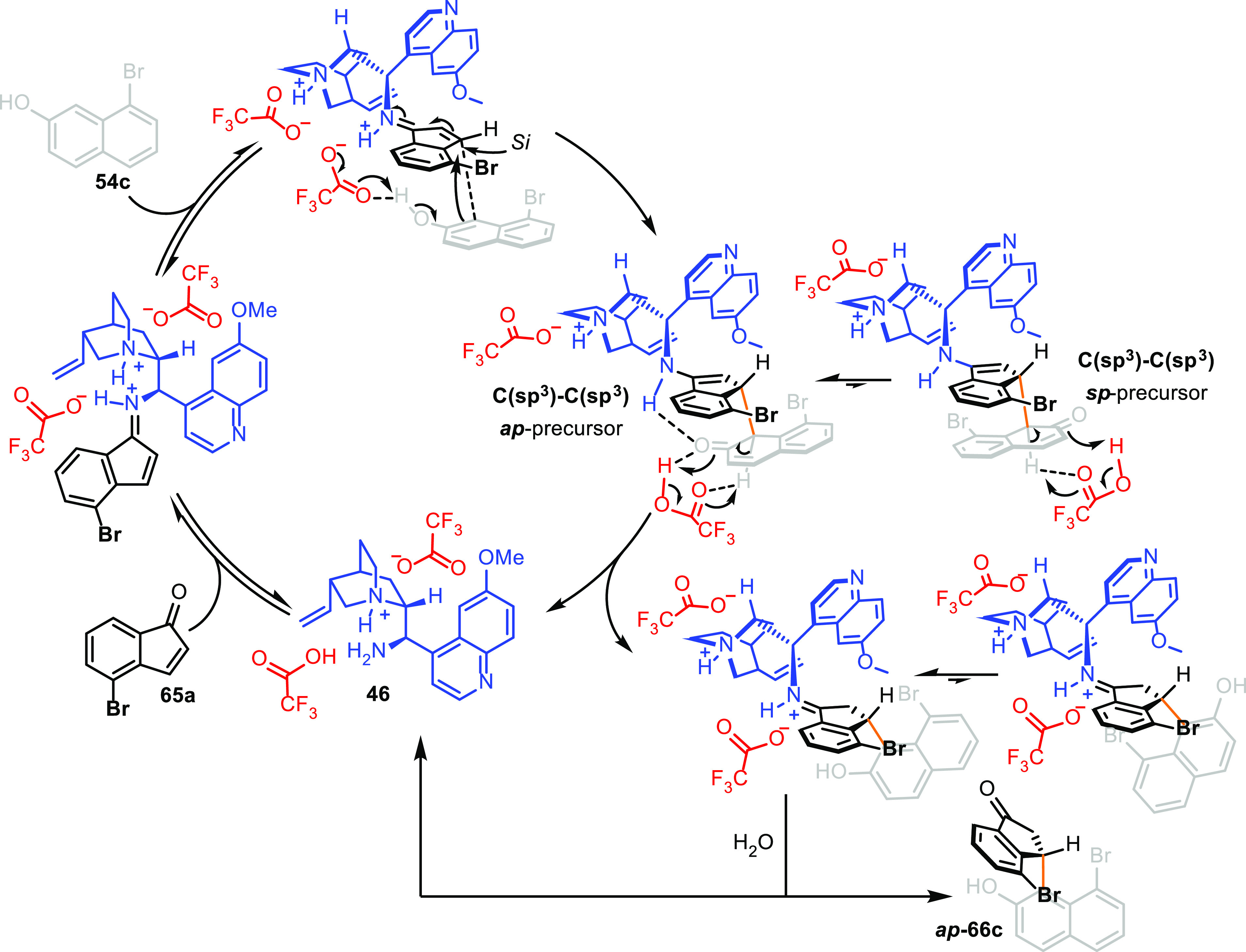
Details of the F–C Alkylation Mechanism

The entire process can be rationalized by understanding the role of the primary amine catalyst **46** and trifluoroacetic acid cocatalyst. We presume that nucleophilic addition of naphthol to the iminium ion is activated by the trifluoroacetate anion, and the resulting C(sp^3^)–C(sp^3^) intermediate is harnessed in a closed rigid geometry that prevents it from free rotation along the new C–C bond. Rapid aromatization furnished the observed major *ap*-conformational diastereoisomer (Scheme 25).

The DFT calculation supports the existence of the sole *ap* conformer, as an energy barrier of >4 kcal/mol separates the *ap* from the *sp* ground states. The rotational energy barrier for interconversion of *ap* to *sp* conformer of compound **66** was 25.7 kcal/mol. This value suggests the existence of alkylated naphthols as axial diastereoisomers, in which a stable C(sp^2^)–C(sp^3^) stereogenic axis connects the two reactive units.

**Scheme 26:**
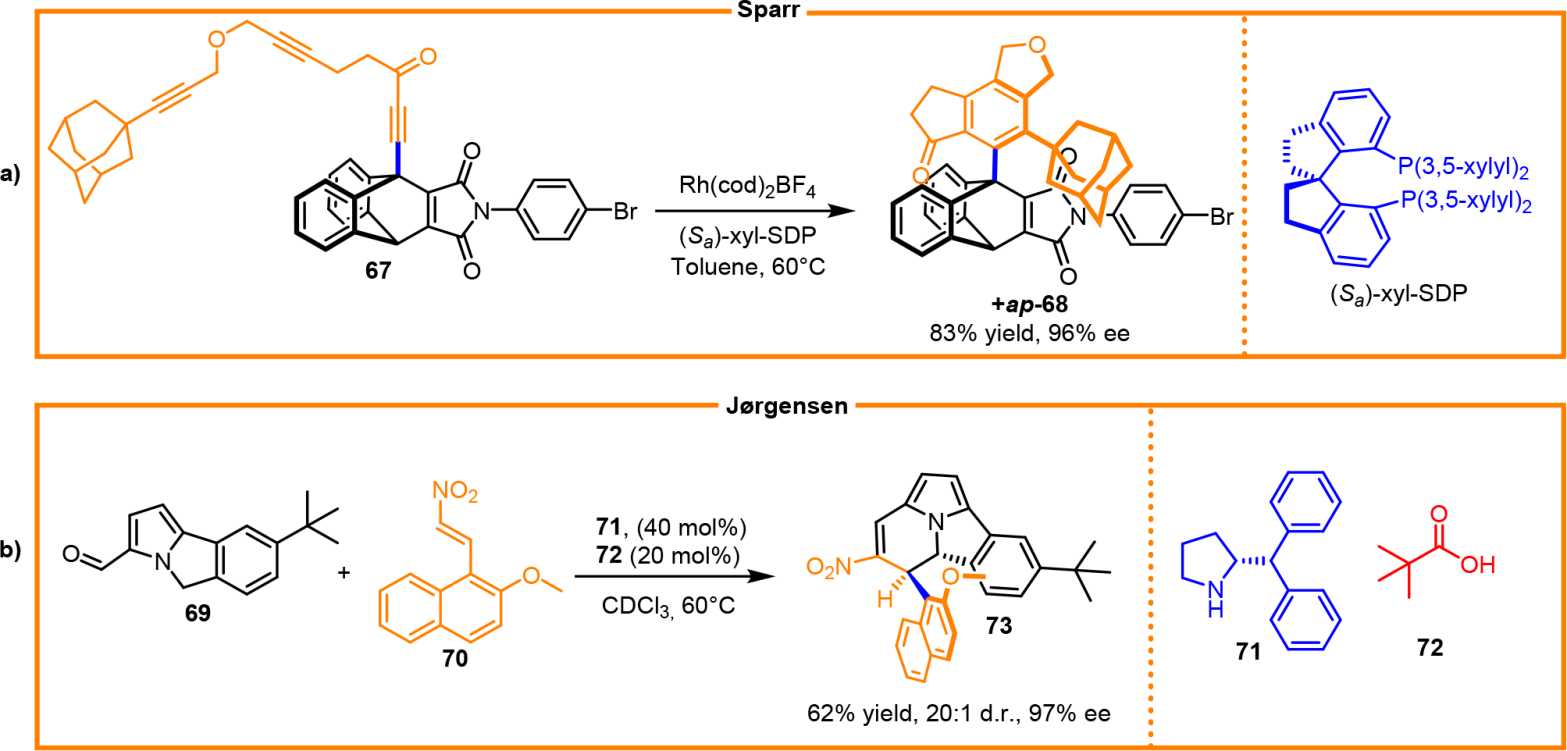
Stereoselective Synthesis of C(sp^2^)–C(sp^3^) Atropisomers

The first enantioselective synthesis of the C(sp^2^)–C(sp^3^) atropisomer was realized by Sparr, who performed efficient Rh-catalyzed [2 + 2 + 2] cyclotrimerization, controlling the formation of more than six stereoisomers of the sole *ap*-triptycene derivative (Scheme 26a).^40^ The enantio- and diastereoselective synthesis of atropisomeric cyclo[3.2.2]azines was realized by Jørgensen et al. (Scheme 26b).^41^ The reaction represents a rare case of the kinetic control of the C(sp^2^)–C(sp^3^) stereogenic axis, providing access to new atropisomeric cyclazine scaffolds.

## 5 Enantio- and Diastereoselective Synthesis of N–N Atropisomers

**Scheme 27:**
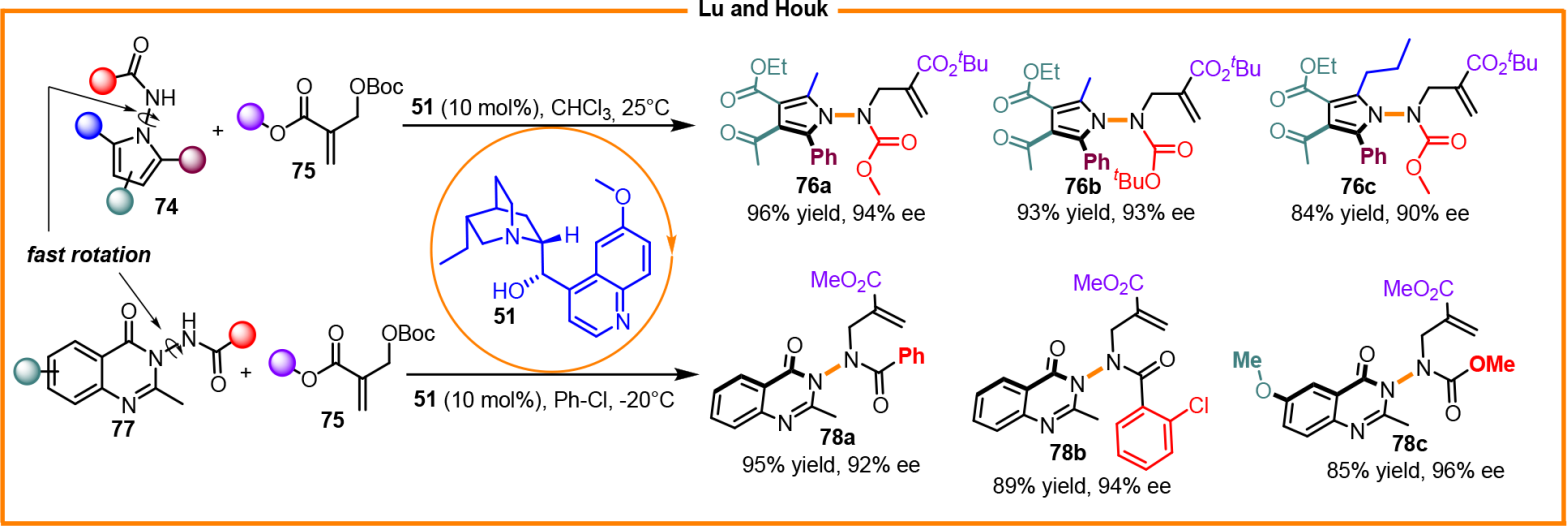
Atropselective Synthesis of 1-Aminopyrroles and 3-Quinazolinones

The constant search for atropisomers that differ from the classical C–C or C–X framework, where X is O, S, and N, has prompted chemists to explore novel reactions and strategies. Recently, several research groups have turned their attention toward a relatively new class of atropisomers containing rotationally impeded N–N single bonds. The existence of N–N atropisomers has long been known from structural studies on the stereoelectronic properties of molecules containing N–N single bonds^42^ and the isolation of bioactive natural products.^43^ Moreover, application of 2,2′-bis(diphenylphosphino)-1,1′-bibenzimidazole as ligands for asymmetric synthesis^44^ and use of 9,9′-bicarbazole derivatives as functional materials^45^ contributed to recognizing the importance of N–N atropisomers. However, the enantioselective construction of N–N atropisomers is yet to be explored. In 2018, Rinaldi et al. reported the first stable atropisomeric hydrazide as an intermediate in the preparation of isosteres of amino acids and conformationally restricted γ-lactams.^46^ Moreover, Lu and Houk et al. realized the synthesis of 1-aminopyrroles and 3-aminoquinazolinones through dynamic kinetic resolution using dihydroquinidine **51** as the catalyst, enabling nitrogen alkylation using Bayliss–Hilmann carbonate (**76a–c** and **78a–c**) (Scheme 27).^47^

**Scheme 28:**
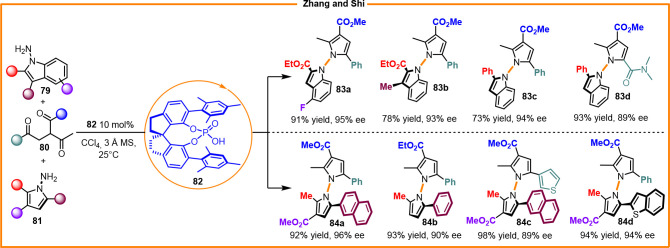
Enantioselective Synthesis of Indole-Pyrrole and 1,1′-Pyrrole Atropisomers

Liu and Lu et al. synthesized atropisomeric pyrroles through FC alkylation using a Cu-bisoxazoline catalyst and arylation of pyrroles using diaryliodonium salt promoted by a Cu-bis(phosphine) dioxide catalyst.^48^ Li et al. realized the *N*-acylation and *N*-alkylation reactions of quinazolinone type benzamide,^49^ and Zhang and Shi et al. performed the chiral phosphoric acid catalyzed synthesis of N–N axially chiral indoles (**83a**–**d**) and pyrroles (**84a**–**d**) (Scheme 28).^50^

A similar approach has been realized by Zhao and Yang et al., who reported the enantiodivergent Paal–Knorr reaction to produce 1,1′-bipyrroles.^51^

**Scheme 29:**
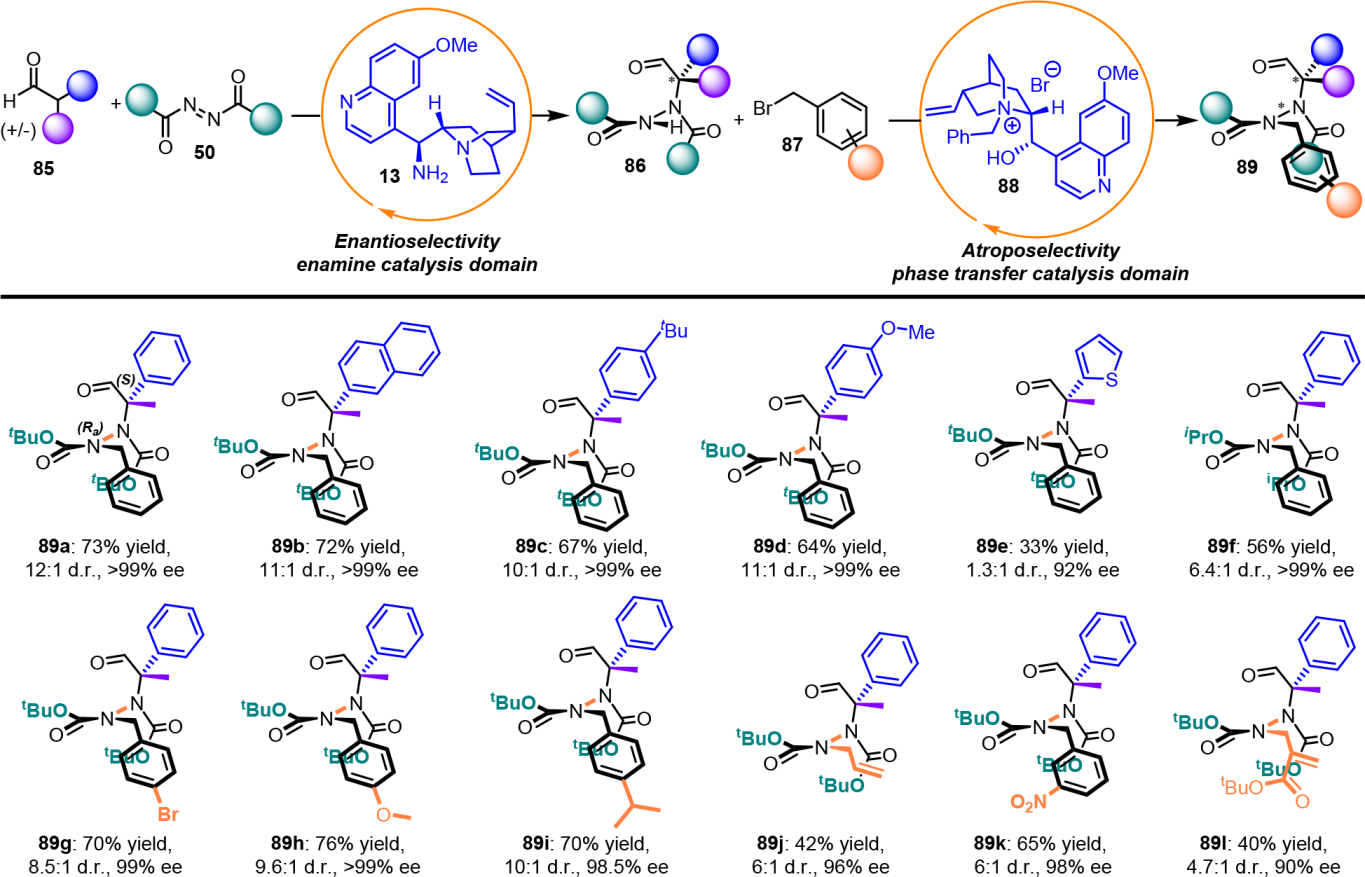
Atropselective Synthesis of Hydrazides

Owing to the importance of N–N atropisomers and our interest in the search for novel strategies for synthesis of atropisomers, we performed the first catalytic stereoselective synthesis of hydrazides containing a rotationally stable N–N single bond.^3^ Our catalytic strategy was based on the use of azodicarboxylate derivatives as a precursor of the N–N single bond. At the design stage, we reasoned that azodicarboxylates can act as reagents in two catalytic reactions that sequentially work together to increase the steric hindrance around the N–N single bond, affording rotationally stable chiral tetrasubstituted hydrazides. To pursue our aim, we selected amination of racemic α-branched aldehydes **85** using 9-*epi*-9-amino-9-deoxyquinine **13** primary amine as the catalyst and the N-alkylation of the resulting trisubstituted hydrazide **86** using the commercially available quinidinium bromide salt **88** (Scheme 29).

**Scheme 30:**
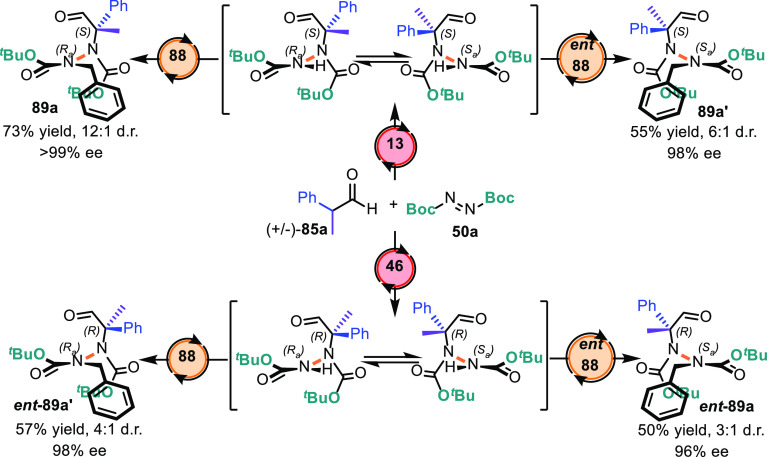
Stereodivergent Synthesis of Atropisomeric Hydrazides via Catalyst Permutation

The asymmetric synthesis of atropisomeric hydrazides performed using a one-pot protocol is applicable to a large series of branched aldehydes and benzyl bromides bearing different substituents (**89a**–**l**). This process furnishes a high level of enantioselectivity and diastereoselectivity and can be extended to different electrophiles, such as allyl iodide (**89j**) and Morita–Baylis–Hillman carbonate (**89l**) albeit with poor yields and diastereoselectivity. The *N*-benzylquinidinium bromide salt **88** exhibited good control of the stereogenic axis, revealing a preference for the *R*_*a*_ configuration. The rotational energy barrier was experimentally determined as 28.3 kcal/mol for compound **89a**; however, hydrazide intermediate **86** is presumably a transient atropisomeric molecule possessing a calculated rotational energy barrier of 20.6 kcal/mol. Interestingly, we investigated the possibility to realize stereodivergent synthesis that provides access to stereoisomers based on a simple catalyst permutation (Scheme 30). Enantiocontrol was well maintained, and diastereoselectivity was not considerably high owing to a matched/mismatched effect between the PT catalyst combination and stereocenter of the tertiary hydrazide intermediate. When low enantiocontrol was observed in the amination reaction using catalyst **46**, i.e., the quasi-enantiomer of catalyst **13** (96% ee vs 65% ee), diatsereoselectivity was low in the resulting tetrasubstituted hydrazide.

**Scheme 31:**
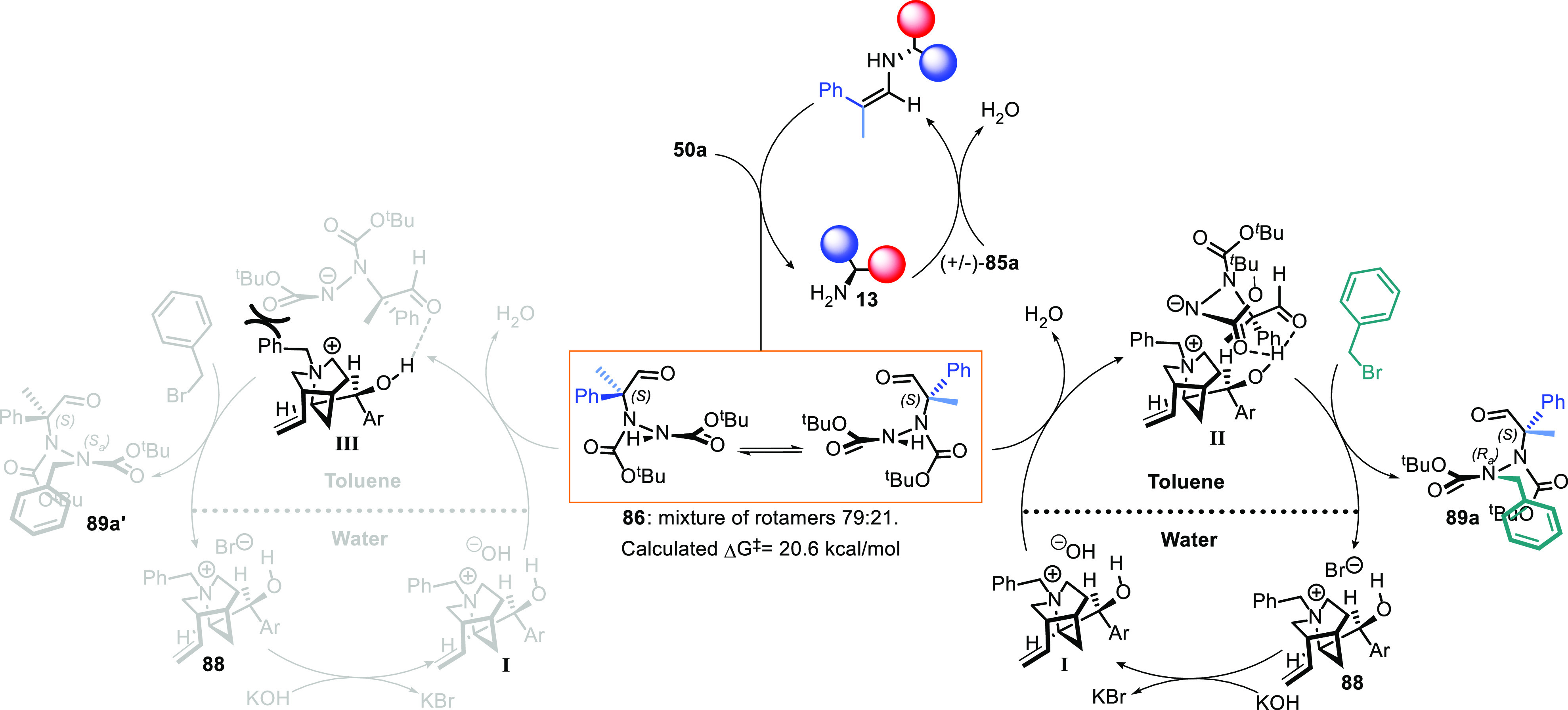
Proposed Mechanism for the Synthesis of Atropisomeric Hydrazides

The reaction mechanism has not been deeply investigated, particularly the atropselective alkylation step. However, we believe that the bifunctional nature of the PT catalyst **88** is fundamental for determining the stereogenic axis configuration observed. The hydroxide anion of **I** deprotonates the intermediate hydrazide, and the *N*-benzylquinidinium cation simultaneously interacts with the negatively charged nitrogen atom via ion pairing and with the carbonylic oxygen atoms via hydrogen bond-forming intermediate **II**. As shown in Scheme 31, the interactions preferentially occur on the hydrazide anion precursor of the final configuration because a compact associated species is formed.

## 6 Conclusion and Outlook

We have presented our findings on the synthesis of different classes of atropisomers focusing on the use of cinchona alkaloid derivatives as organic catalysts. The obtained results reinforce the strategic position occupied by organocatalysis in enantioselective preparation of C–N, C–C, and N–N axially chiral compounds. New enantioselective transformations that furnish high molecular complexity can be realized using accessible catalysts prepared from commercially available sources. In most transformations highlighted, the activity of the cinchona organocatalyst revealed is comparable with those of active metal species using covalent and noncovalent activation modes. This is also the case with olefination via the Knoevenagel reaction and Friedel–Craft-type alkylation of naphthols, in which addition to an iminium ion intermediate occurs efficiently using bulky reaction partners under mild reaction conditions. Furthermore, another important feature is the ability of cinchona organic catalysts, such as primary amines or organic bases, to efficiently differentiate the enantiotopic faces of pro-atropchiral substrates, exploiting their ability to realize fruitful secondary interaction (π-stacking, hydrogen bonds, ion pairing) or to be part of a strong catalytic cooperation with acidic cocatalysts, similar to the case of vinylogous desymmetrization of maleimides. We have focused on the development of novel atropselective reactions; it prompted us to explore new fields and molecular complexity as demonstrated by the recent results of the synthesis of N–N atropisomers, where an efficient one-pot sequential relay catalysis was conducted using two different activation modes based on the use of a primary amine and PT catalyst. Although we have demonstrated that cinchona catalysts can be largely employed in the synthesis of molecules containing stereogenic axes and centers, the catalytic efficiency and versatility can be certainly improved. For these reasons, novel catalysts, enabling the use of cinchona alkaloid derivatives in industrial applications or to achieve a novel family of atropisomers, are highly required.

## References

[ref1] Di Iorio N.; Righi P.; Mazzanti A.; Mancinelli M.; Ciogli A.; Bencivenni G. Remote Control of Axial Chirality: Aminocatalytic Desymmetrization of *N*-Arylmaleimides via Vinylogous Michael Addition. J. Am. Chem. Soc. 2014, 136, 10250 10.1021/ja505610k. 25006984

[ref2] Crotti S.; Di Iorio N.; Artusi C.; Mazzanti A.; Righi P.; Bencivenni G. Direct Access to Alkylideneoxindoles via Axially Enantioselective Knoevenagel Condensation. Org. Lett. 2019, 21, 3013 10.1021/acs.orglett.9b00505. 30977662

[ref3] Portolani C.; Centonze G.; Luciani S.; Pellegrini A.; Righi P.; Mazzanti A.; Ciogli A.; Sorato A.; Bencivenni G. Synthesis of Atropisomeric Hydrazides by One-Pot Sequential Enantio- and Diastereoselective Catalysis. Angew. Chem., Int. Ed. 2022, 61, e202209895 10.1002/anie.202209895. PMC9826270 36036383

[ref4] aChen Y.; Yekta S.; Yudin A. K. Modified BINOL Ligands in Asymmetric Catalysis. Chem. Rev. 2003, 103, 3155 10.1021/cr020025b. 12914495

[ref5] Bringmann G.; Gulder T.; Gulder T. A. M.; Breuning M. Atroposelective Total Synthesis of Axially Chiral Biaryl Natural Products. Chem. Rev. 2011, 111, 563 10.1021/cr100155e. 20939606

[ref6] Cheng J. K.; Xiang S. H.; Li S.; Ye L.; Tan B. Recent Advances in Catalytic Asymmetric Construction of Atropisomers. Chem. Rev. 2021, 121, 4805 10.1021/acs.chemrev.0c01306. 33775097

[ref7] Bringmann G.; Price Mortimer A. J.; Keller P. A.; Gresser M. J.; Garner J.; Breuning M. Atroposelective Synthesis of Axially Chiral Biaryl Compounds. Angew. Chem., Int. Ed. 2005, 44, 5384 10.1002/anie.200462661. 16116589

[ref8] aMei G.-J.; Koay W. L.; Guan C.-Y.; Lu Y. Atropisomers Beyond the C-C Axial Chirality: Advances in Catalytic Asymmetric Synthesis. Chem. 2022, 8, 1855 10.1016/j.chempr.2022.04.011.

[ref9] Carmona J. A.; Rodriguez-Franco C.; Fernandez R.; Hornillos V.; Lassaletta J. M. Atroposelective Transformation of Axially Chiral (Hetero)Biaryls. From Desymmetrization to Modern Resolution Strategies. Chem. Soc. Rev. 2021, 50, 2968 10.1039/D0CS00870B. 33491680

[ref10] Di Iorio N.; Crotti S.; Bencivenni G. Organocatalytic Desymmetrization Reactions for the Synthesis of Axially Chiral Compounds. Chem. Rec. 2019, 19, 2095 10.1002/tcr.201800194. 30730097

[ref11] Mori K.; Ichikawa Y.; Kobayashi M.; Shibata Y.; Yamanaka M.; Akiyama T. Enantioselective Synthesis of Multisubstituted Biaryl Skeleton by Chiral Phosphoric Acid Catalyzed Desymmetrization/Kinetic Resolution Sequence. J. Am. Chem. Soc. 2013, 135, 3964 10.1021/ja311902f. 23413828

[ref12] Armstrong R. J.; Smith M. D. Catalytic Enantioselective Synthesis of Atropisomeric Biaryls: A Cation-Directed Nucleophilic Aromatic Substitution Reaction. Angew. Chem., Int. Ed. 2014, 126, 13036 10.1002/ange.201408205. 25257677

[ref13] Chauhan P.; Kaur J.; Chimni S. S. Asymmetric Organocatalytic Addition Reactions of Maleimides: A Promising Approach Towards the Synthesis of Chiral Succinimide derivatives. Chem.—Asian J. 2013, 8, 328 10.1002/asia.201200684. 22997193

[ref14] aDe Figueiredo R. M.; Fröhlich R.; Christmann M. Efficient Synthesis and Resolution of Pyrrolizidines. Angew. Chem., Int. Ed. 2007, 119, 2941 10.1002/ange.200605035. 17340649

[ref15] Curran D. P.; Qi H.; Geib S. J.; DeMello N. C. Atroposelective Thermal Reactions of Axially Twisted Amides and Imides. J. Am. Chem. Soc. 1994, 116, 3131 10.1021/ja00086a056.

[ref16] Tampellini N.; Righi P.; Bencivenni G. Computational Investigation on the Origin of Atroposelectivity for the Cinchona Alkaloid Primary Amine-Catalyzed Vinylogous Desymmetrization of *N*-(2-*t*-Butylphenyl) Maleimides. J. Org. Chem. 2021, 86, 11782 10.1021/acs.joc.1c01235. 34347451 PMC8764656

[ref17] Bartoli G.; Bosco M.; Carlone A.; Cavalli A.; Locatelli M.; Mazzanti A.; Ricci P.; Sambri L.; Melchiorre P. Organocatalytic Asymmetric Conjugate Addition of 1, 3-Dicarbonyl Compounds to Maleimides. Angew. Chem., Int. Ed. 2006, 45, 4966 10.1002/anie.200600370. 16819743

[ref18] Di Iorio N.; Champavert F.; Erice A.; Righi P.; Mazzanti A.; Bencivenni G. Targeting Remote Axial Chirality Control of *N*-(2-*Tert*-Butylphenyl) Succinimides by Means of Michael Addition Type Reactions. Tetrahedron 2016, 72, 5191 10.1016/j.tet.2016.02.052.

[ref19] aVintonyak V. V.; Warburg K.; Kruse H.; Grimme S.; Hübel K.; Rauh D.; Waldmann H. Identification of Thiazolidinones Spiro-fused to Indolin-2-Ones as Potent and Selective Inhibitors of the Mycobacterium Tuberculosis Protein Tyrosine Phosphatase B. Angew. Chem., Int. Ed. 2010, 49, 5902 10.1002/anie.201002138. 20632348

[ref20] Li L.; Chen W.; Yang W.; Pan Y.; Liu H.; Tan C. H.; Jiang Z. Bicyclic Guanidine-Catalyzed Asymmetric Michael Additions of 3-Benzyl-Substituted Oxindoles to *N*-Maleimides. Chem. Commun. 2012, 48, 5124 10.1039/c2cc31587d. 22516982

[ref21] Di Iorio N.; Soprani L.; Crotti S.; Marotta E.; Mazzanti A.; Righi P.; Bencivenni G. Michael Addition of Oxindoles to *N*-(2-*Tert*-Butylphenyl)Maleimides: Efficient Desymmetrization for the Synthesis of Atropisomeric Succinimides with Quaternary and Tertiary Stereocenters. Synthesis 2017, 49, 1519 10.1055/s-0036-1588408.

[ref22] Merino P.; Marqués-López E.; Tejero T.; Herrera R. P. Enantioselective Organocatalytic Diels-Alder Reactions. Synthesis 2010, 1 10.1055/s-0029-1217130.

[ref23] Riant O.; Kagan H. B. Asymmetric Diels-Alder Reaction Catalyzed by Chiral Bases. Tetrahedron Lett. 1989, 30, 7403 10.1016/S0040-4039(00)70709-X.

[ref24] Wu L.-Y.; Bencivenni G.; Mancinelli M.; Mazzanti A.; Bartoli G.; Melchiorre P. Organocascade Reactions of Enones Catalyzed by a Chiral Primary Amine. Angew. Chem., Int. Ed. 2009, 48, 7196 10.1002/anie.200903280. 19718738

[ref25] Eudier F.; Righi P.; Mazzanti A.; Ciogli A.; Bencivenni G. Organocatalytic Atroposelective Formal Diels–Alder Desymmetrization of *N*-Arylmaleimides. Org. Lett. 2015, 17, 1728 10.1021/acs.orglett.5b00509. 25778786

[ref26] Eliel E. L.; Wilen S. H. In Stereochemistry of Organic Compounds; John Wiley-Interscience: New York, 1994.

[ref27] aDing A.; Meazza M.; Guo H.; Yang J. W.; Rios R. New Development in the Enantioselective Synthesis of Spiro compounds. Chem. Soc. Rev. 2018, 47, 5946 10.1039/C6CS00825A. 29953153

[ref28] Gramigna L.; Duce S.; Filippini G.; Fochi M.; Franchini M.; Bernardi L. Organocatalytic Asymmetric Wittig Reactions: Generation of Enantioenriched Axially Chiral Olefins Breaking a Symmetry Plane. Synlett 2011, 2745 10.1055/s-0031-1289516.

[ref29] Knoevenagel E. Ueber eine Darstellungsweise der Glutarsaure. Ber. Dtsch. Chem. Ges. 1894, 27, 2345 10.1002/cber.189402702229.

[ref30] Lee A.; Michrowska A.; Sulzer-Mosse S.; List B. The Catalytic Asymmetric Knoevenagel Condensation. Angew. Chem., Int. Ed. 2011, 50, 1707 10.1002/anie.201006319. 21308938

[ref31] Liao G.; Zhou T.; Yao Q.-J.; Shi B.-F. Recent Advances in the Synthesis of Axially Chiral Biaryls via Transition Metal-Catalyzed Asymmetric C-H Functionalization. Chem. Commun. 2019, 55, 8514 10.1039/C9CC03967H. 31276136

[ref32] Brandes S.; Bella M.; Kjærsgaard A.; Jørgensen K. A. Chirally Aminated Naphthols–Organocatalytic Synthesis of Non-Biaryl Atropisomers by Asymmetric Friedel-Crafts Amination. Angew. Chem., Int. Ed. 2006, 45, 1147 10.1002/anie.200503042. 16389601

[ref33] Chen Y.-H.; Cheng D.-J.; Zhang J.; Wang Y.; Liu X.-Y.; Tan B. Atroposelective Synthesis of Axially Chiral Biaryldiols via Organocatalytic Arylation of 2-Naphthols. J. Am. Chem. Soc. 2015, 137, 15062 10.1021/jacs.5b10152. 26560999

[ref34] aQi L.-W.; Li S.; Xiang S.-H.; Wang J.; Tan B. Asymmetric Construction of Atropisomeric Biaryls via a Redox Neutral Cross-Coupling Strategy. Nat. Catal. 2019, 2, 314 10.1038/s41929-019-0247-1.

[ref35] aCoombs G.; Sak M. H.; Miller S. J. Peptide-Catalyzed Fragment Couplings That Form Axially Chiral Non-C2-Symmetric Biaryls. Angew. Chem., Int. Ed. 2020, 59, 2875 10.1002/anie.201913563. PMC7002259 31793137

[ref36] Lomas J. S.; Dubois J. E. Conformational Isomerism in o-Tolyldi-*tert*-butylcarbinol. J. Org. Chem. 1976, 41, 3033 10.1021/jo00880a026.

[ref37] aRaut V. S.; Jean M.; Vanthuyne N.; Roussel C.; Constantieux T.; Bressy C.; Bugaut X.; Bonne D.; Rodriguez J. Enantioselective Syntheses of Furan Atropisomers by an Oxidative Central-to-Axial Chirality Conversion Strategy. J. Am. Chem. Soc. 2017, 139, 2140 10.1021/jacs.6b11079. 28106391

[ref38] Di Iorio N.; Filippini G.; Mazzanti A.; Righi P.; Bencivenni G. Controlling the C(sp^3^)–C(sp^2^) Axial Conformation in the Enantioselective Friedel–Crafts-Type Alkylation of β-Naphthols with Inden-1-Ones. Org. Lett. 2017, 19, 6692 10.1021/acs.orglett.7b03415. 29199831

[ref39] Paradisi E.; Righi P.; Mazzanti A.; Ranieri S.; Bencivenni G. Iminium Ion Catalysis: The Enantioselective Friedel–Crafts alkylation–Acetalization Cascade of Naphthols with α,β-Unsaturated Cyclic Ketones. Chem. Commun. 2012, 48, 11178 10.1039/c2cc35582e. 23010910

[ref40] Wu X.; Witzig R. M.; Beaud R.; Fischer C.; Haussinger D.; Sparr C. Catalyst Control Over Sixfold Stereogenicity. Nat. Catal. 2021, 4, 457 10.1038/s41929-021-00615-z.

[ref41] Bertuzzi G.; Corti V.; Izzo J. A.; Ricko S.; Jessen N. I.; Jørgensen K. A. Organocatalytic Enantioselective Construction of Conformationally Stable C(sp^2^)-C(sp^3^) Atropisomers. J. Am. Chem. Soc. 2022, 144, 1056 10.1021/jacs.1c12619. 34990550

[ref42] Verma S. M.; Prasad R. Conformational Analysis by Nuclear Magnetic Resonance Spectroscopy. N’ Derivatives of *N*-Aminocamphorimides. J. Org. Chem. 1973, 38, 1004 10.1021/jo00945a031.

[ref43] Zhang Q.; Mándi A.; Li S.; Chen Y.; Zhang W.; Tian X.; Zhang H.; Li H.; Zhang W.; Zhang S.; Ju J.; Kurtán T.; Zhang C. N–N-Coupled Indolo-Sesquiterpene Atropo-Diastereomers from a Marine-Derived Actinomycete. Eur. J. Org. Chem. 2012, 5256 10.1002/ejoc.201200599.

[ref44] Benincori T.; Brenna E.; Sannicolò F.; Trimarco L.; Antognazza P.; Cesarotti E.; Demartin F.; Pilati T.; Zotti G. Chiral Atropisomeric Five-Membered Biheteroaromatic Diphosphines: New ligands of the Bibenzimidazole and Bisindole Series. J. Organomet. Chem. 1997, 529, 445 10.1016/S0022-328X(96)06682-X.

[ref45] Liu X.-Y.; Zhang Y.-L.; Fei X.; Liao L.-S.; Fan J. 9,9′-Bicarbazole: New Molecular Skeleton for Organic Light-Emitting Diodes. Chem.—Eur. J. 2019, 25, 4501 10.1002/chem.201806314. 30684362

[ref46] Amabili P.; Amici A.; Campisi G.; Guerra G.; Monari M.; Orena M.; Piccinelli F.; Rinaldi S.; Tolomelli A. Synthesis of Enantipoure Isostere of Amino Acids Containing a Quaternary Stereocenter: Experimental and Computational Evaluation of a Novel Class of Atropisomers. Eur. J. Org. Chem. 2018, 6524 10.1002/ejoc.201801213.

[ref47] Mei G.-J.; Wong J. J.; Zheng W.; Nangia A. A.; Houk K. N.; Lu Y. Rational Design and Atroposelective Synthesis of N–N Axially Chiral Compounds. Chem. 2021, 7, 2743 10.1016/j.chempr.2021.07.013.

[ref48] aWang X.- M.; Zhang P.; Xu Q.; Guo C.-Q.; Zhang D.-B.; Lu C.-J.; Liu R.-R. Enantioselective Synthesis of Nitrogen–Nitrogen Biaryl Atropisomers via Copper-Catalyzed Friedel–Crafts Alkylation Reaction. J. Am. Chem. Soc. 2021, 143, 15005 10.1021/jacs.1c07741. 34496212

[ref49] Lin W.; Zhao Q.; Li Y.; Pan M.; Yang C.; Yang G.; Li X. Asymmetric Synthesis of N-N Axially Chiral Compounds via Organocatalytic Atroposelective *N*-Acylation. Chem. Sci. 2021, 13, 141 10.1039/D1SC05360D. 35059162 PMC8694391

[ref50] Chen K.-W.; Chen Z.-H.; Yang S.; Wu S.-F.; Zhang Y.-C.; Shi F. Organocatalytic Atroposelective Synthesis of N-N Axially Chiral Indoles and Pyrroles by De Novo Ring Formation. Angew. Chem., Int. Ed. 2022, 61, e202116829 10.1002/anie.202116829. 35080808

[ref51] Gao Y.; Wang L.-Y.; Zhang T.; Yang B.-M.; Zhao Y. Atroposelective Synthesis of 1,1’-Bipyrroles Bearing a Chiral N-N Axis: Chiral Phosphoric Acid Catalysis with Lewis Acid Induced Enantiodivergence. Angew. Chem., Int. Ed. 2022, 61, e202200371 10.1002/anie.202200371. 35174596

